# Systematic Investigation of Transient Thermo-Fluid-Mass Coupling in a Hydrogen Knudsen Compressor Under Diverse Thermal Non-Equilibrium Waveforms

**DOI:** 10.3390/mi17091021

**Published:** 2026-08-27

**Authors:** Shuhan Chen, Qianhao Xiao, Biyuan Tan, Muyan Cao

**Affiliations:** School of Energy and Power Engineering, Changsha University of Science and Technology, Changsha 410114, China; 202306100215@csust.edu.cn (S.C.); 17773401331@163.com (B.T.); 202306120112@stu.csust.edu.cn (M.C.)

**Keywords:** hydrogen, microfluidics, Knudsen compressor, thermal non-equilibrium, thermal transpiration flow, thermo-fluid coupling

## Abstract

Frequent heat-source drift and environmental disturbances keep hydrogen compressors in a state of thermal non-equilibrium, where transient interactions between thermal transpiration and Poiseuille flows significantly influence system stability and safety. The mechanisms by which time-dependent temperature patterns modulate this coupling remain insufficiently understood. This research addresses this knowledge gap by numerically solving slip-boundary Navier–Stokes equations for six periodic temperature waveforms: rectangular, segmented, square, Gaussian, triangular, and sinusoidal. The results indicate that the heating rate predominantly determines the intensity of forward Poiseuille flow, whereas cooling rate and plateau duration exert comparatively minor effects. Extending the high-temperature plateau from 0.1 to 0.5 s increases both thermal transpiration and backward Poiseuille peaks by more than 100 percent, while slower heating reduces forward Poiseuille peaks by up to 11 percent. Among the six waveforms, the square wave yields the highest peaks for all three flow components. These findings elucidate how waveform structures modulate transient flow responses and offer quantitative guidance for stability assessment and thermal management in hydrogen Knudsen compressors.

## 1. Introduction

The hydrogen Knudsen compressor (HKC) [1,2,3] is distinguished by its lack of moving parts, compact design, and high reliability, making it highly suitable for applications such as fuel recirculation in hydrogen fuel cell vehicles and miniature cryogenic cooling. The HKC operates based on the thermal transpiration effect [4,5,6], which arises from a temperature gradient along the microchannel walls and drives hydrogen transport from the cold end to the hot end. During operation, an opposing Poiseuille flow [7], generated by the pressure gradient, also develops within the channel. The interaction between the Poiseuille flow and the thermal transpiration stream produces a complex, nonlinear thermo-fluid-mass coupling structure [8]. Under unsteady operating conditions, including frequent load fluctuations, heat-source temperature drift, and substantial ambient temperature disturbances, the transient competition between these two flows generates multiscale flow pulsations and intermittent backflow [9,10,11]. This highly unsteady behavior alters local pressure and velocity distributions and can induce abrupt temperature rises and stress concentrations [12,13], which directly threaten the transport stability of the compressor and the safety margins of hydrogen systems. Comprehensive understanding of the transient evolution and coupling mechanisms of Poiseuille flow and thermal transpiration flow under various thermal non-equilibrium processes is therefore a critical scientific prerequisite for ensuring safe, efficient, and stable compressor operation. Non-uniformity and transient variations in the temperature field govern both the magnitude and direction of the thermal transpiration driving force [14,15], as well as the thermal stress distribution in materials and the thermodynamic safety margin of hydrogen. Consequently, focused investigation of internal temperature dynamics is crucial for elucidating multiphysics coupling mechanisms and optimizing thermal management strategies.

Previous research on hydrogen Knudsen compressors has primarily addressed structural optimization and steady-state performance under constant operating conditions. Investigators have systematically varied microchannel geometric parameters, such as aspect ratio [1] and cross-sectional shape [16,17,18], as well as obstacle arrangements and the number of series-connected stages, to analyze the evolution of Knudsen layer distribution, velocity fields, and pressure-rise characteristics. These studies have established first-order mapping relationships between dimensionless parameters and key flow components through dimensional analysis [19]. Ye et al. [20] demonstrated a high pressure-ratio output from a monolithically integrated multistage compressor, thereby confirming the feasibility of the structural design. Such steady-state investigations have provided essential reference points for compressor configuration and baseline performance prediction.

However, in practical service environments, factors such as frequent load fluctuations, heat-source temperature drift, and wide ambient temperature disturbances result in prolonged transient evolution toward thermal non-equilibrium. Lan et al. [21] developed a model of a hydrogen Knudsen compressor with sawtooth-shaped microchannels and identified thermal expansion flow, thermal transpiration flow, and Poiseuille flow as the primary factors influencing thermal non-equilibrium evolution. Xiao et al. [7] applied proper orthogonal decomposition to identify coherent structures within the hydrogen flow field during non-equilibrium evolution. The results indicate that the magnitude of the temperature gradient is the dominant mechanism governing the thermal non-equilibrium process. Despite these advances, most studies on thermal non-equilibrium have focused on evolution under specific thermal boundary conditions [22,23,24]. There remains a lack of systematic comparative investigation into how different waveforms of temperature-gradient loading affect the transient coupling characteristics between thermal transpiration flow and Poiseuille flow.

This study systematically investigates transient thermo-fluid-mass coupling in a hydrogen Knudsen compressor (HKC) under six representative periodic temperature waveforms: rectangular, segmented, square, Gaussian, triangular, and sinusoidal. Previous research has primarily addressed steady-state performance or transient evolution under single thermal boundary conditions. However, the comparative effects of distinct waveform structures, particularly their heating and cooling rates and plateau characteristics, on the transient competition among the three flow components (forward Poiseuille flow, backward Poiseuille flow, and wall thermal transpiration flow) remain unexplored. To address this gap, a control-variable methodology is employed to isolate and quantify the independent contributions of heating rate, cooling rate, and high-temperature plateau residence time to each flow state, establishing their respective contribution weights and effective bounds for the first time. The results reveal the underlying mechanism by which thermal transpiration drives pressure-gradient reversal through mass redistribution. Furthermore, these findings provide a quantitative basis for evaluating flow stability, optimizing thermal management strategies, and defining safety boundaries for hydrogen Knudsen compressors subjected to wide-range thermal disturbances.

## 2. Numerical Simulation Methods

**Figure 1:**
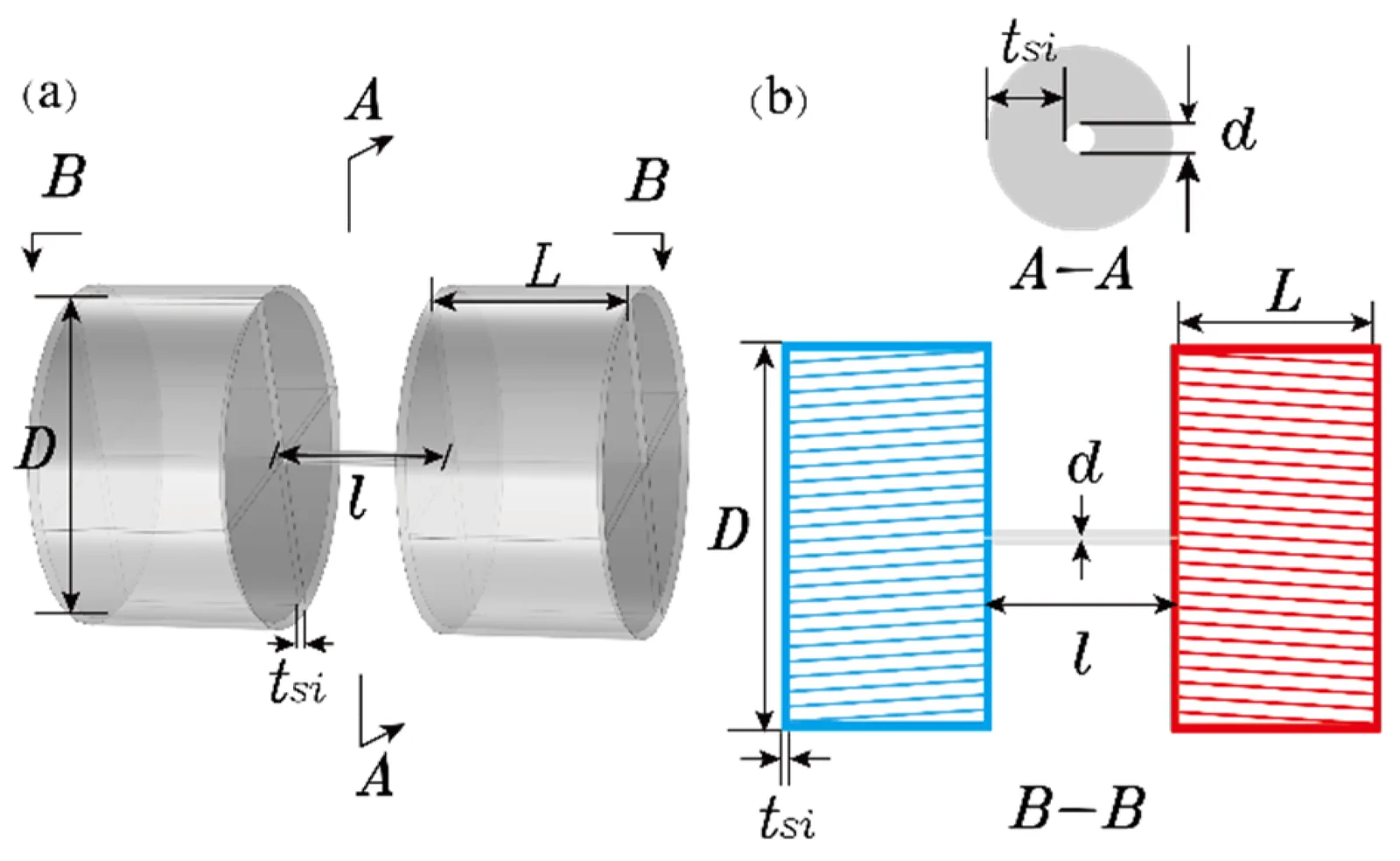
Geometric schematic of the hydrogen Knudsen compressor: (**a**) Three-dimensional configuration; (**b**) two-dimensional cross section.

This study examines the three-dimensional structure (Figure 1) and the two-dimensional cross-section of the hydrogen Knudsen compressor. The compressor comprises cold and hot hydrogen reservoirs connected by a cylindrical microchannel, with a silicon layer of specified thickness deposited on the surfaces of both the reservoirs and the channel. Table 1 lists the relevant compressor parameters. The temperature in the hot hydrogen reservoir varies periodically over time, facilitating the investigation of non-equilibrium evolution processes under varying thermal conditions. Monitoring pressure and velocity variations along both the centreline and the perpendicular centreline of the microchannel reveal the dominant characteristics of hydrogen flow during the non-equilibrium process.

### 2.1. Governing Equations and Boundary Conditions

The hydrogen flow and energy exchange in the hydrogen Knudsen compressor are governed by the incompressible Navier–Stokes (NS) equations and the energy conservation equation [25,26,27].

(1) $$\begin{array}{l} \frac{\partial \rho }{\partial t}+\frac{\partial \left(\rho u_{i}\right)}{\partial x_{i}}=0\\ \frac{\partial \left(\rho u_{i}\right)}{\partial t}+\frac{\partial \left(\rho u_{i}u_{j}\right)}{\partial x_{j}}=-\frac{\partial p}{\partial x_{i}}+\frac{\partial \tau_{ij}}{\partial x_{j}}\\ \frac{\partial \left(\rho e\right)}{\partial t}+\frac{\partial \left(\rho u_{i}e\right)}{\partial x_{i}}=-p\frac{\partial u_{i}}{\partial x_{i}}+\tau_{ij}\frac{\partial u_{i}}{\partial x_{j}}-\frac{\partial q_{i}}{\partial x_{i}} \end{array}$$

where the symbols *u*, *ρ*, *p*, *τ_ij_*, and *t* represent the velocity, density, pressure, stress, and time of hydrogen within the channel, respectively. The symbols *e* and *q* represent the internal energy and total heat flux of hydrogen, respectively.

The Knudsen number is defined as Kn = *λ*/*d*, where *λ* is the molecular mean free path of hydrogen and *d* is the diameter of the microchannel [1]. Based on the definition of the Knudsen number, the value within the microchannel of the hydrogen Knudsen compressor examined in this study ranges from 0.01 to 0.1. This range indicates that the hydrogen flow operates in the slip-flow regime, necessitating the application of slip-flow boundary conditions [28,29,30] on the microchannel walls.

(2) $$u_{slip}=\sigma_{s}\frac{\lambda }{\mu }\left(\tau n-\left(n^{T}\tau n\right)n\right)+\sigma_{T}\frac{\mu }{\rho T_{g}}\left(\nabla T_{w}-\left(n\cdot \nabla T_{w}\right)n\right)$$

where **n** and *τ* denote the normal and viscous stress tensors on the wall, respectively, while *λ* and *μ* represent the mean free path and viscosity of hydrogen. The temperature slip coefficient, *σ_T_*, is treated as a constant with a value of 0.75 [21] in this study. The viscous slip coefficient *σ_s_* is defined as (2 − *α_t_*)/*α_t_* [2]. *α_t_* is the tangential momentum accommodation coefficient, quantifying the degree of diffuse reflection of molecules from the wall. *α_t_* is set to 0.9 [30] in this work. *T_w_* and *T_g_* refer to the temperatures of the channel wall and the hydrogen gas, respectively.

**Figure 2:**
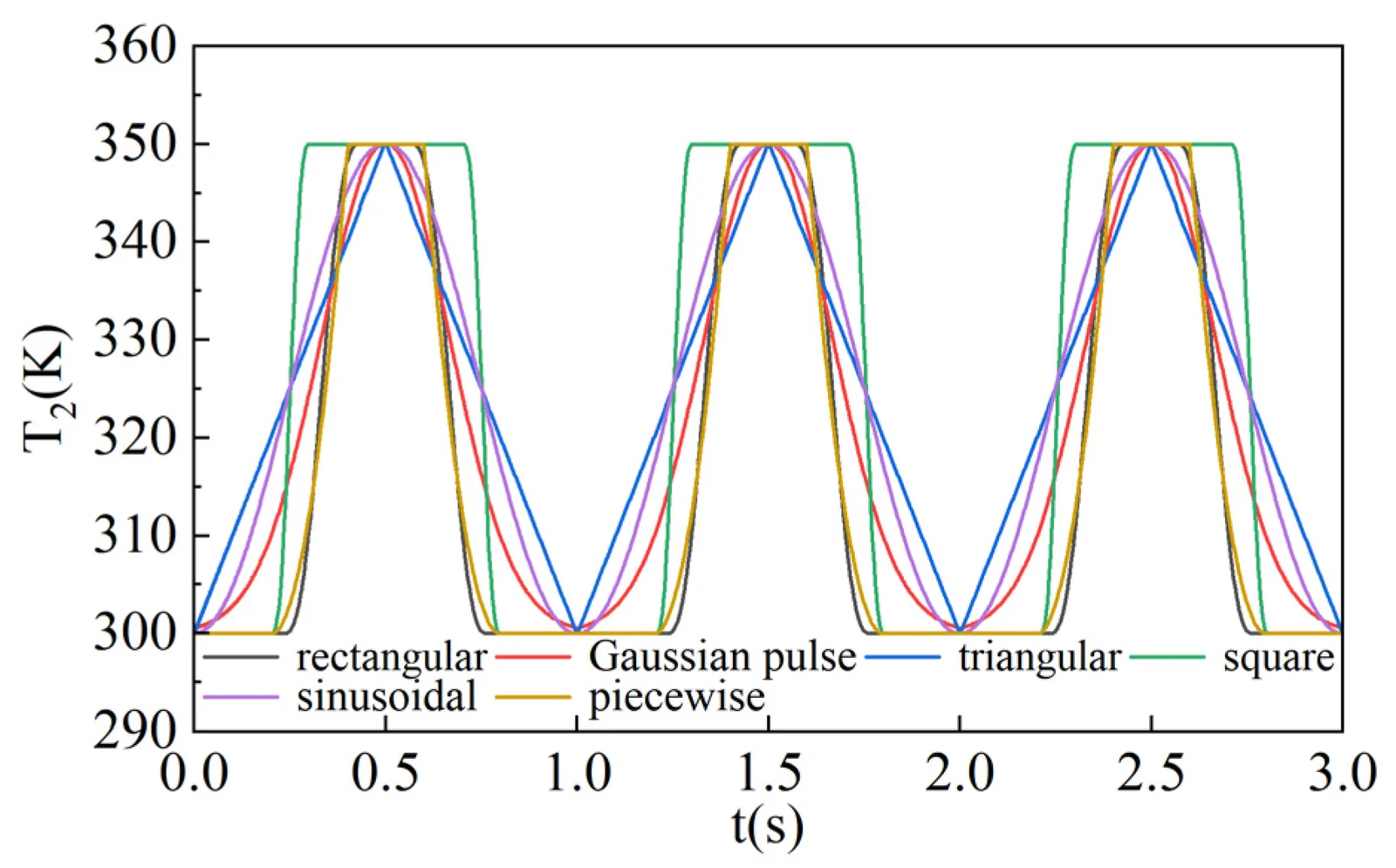
Temperature evolution of the hot reservoir over time for selected cases.

Figure 2 illustrates that the temperature difference between the reservoirs at both ends establishes a temperature gradient along the microchannel wall, inducing a thermal transpiration effect. This effect drives wall slip and generates a thermal transpiration flow from the cold reservoir toward the hot reservoir. The temperature *T*_1_ of the cold reservoir remains constant, while the temperature of the hot reservoir varies periodically over time. The temperature of the hot-side reservoir is regulated by a polyimide heating film, and heat is conducted through the silicon material to the inner wall, where it subsequently heats the hydrogen gas. Six distinct temperature variation patterns are analyzed, each completing three cycles within 3 s. To minimize the influence of external conditions on the temperature gradient within the microchannel, the silicon material surrounding the microchannel wall is assigned an adiabatic boundary condition. The lower-left corner of the cold reservoir serves as a pressure reference point with a fixed gauge pressure *P*_0_ = 0 Pa, which ensures closure of the governing equations and stable numerical convergence throughout the simulation. This setting serves only as a benchmark for the numerical solution and does not imply that the physical pressure at that location is an absolute vacuum.

### 2.2. Mesh Independence and Reliability Validation

**Figure 3:**
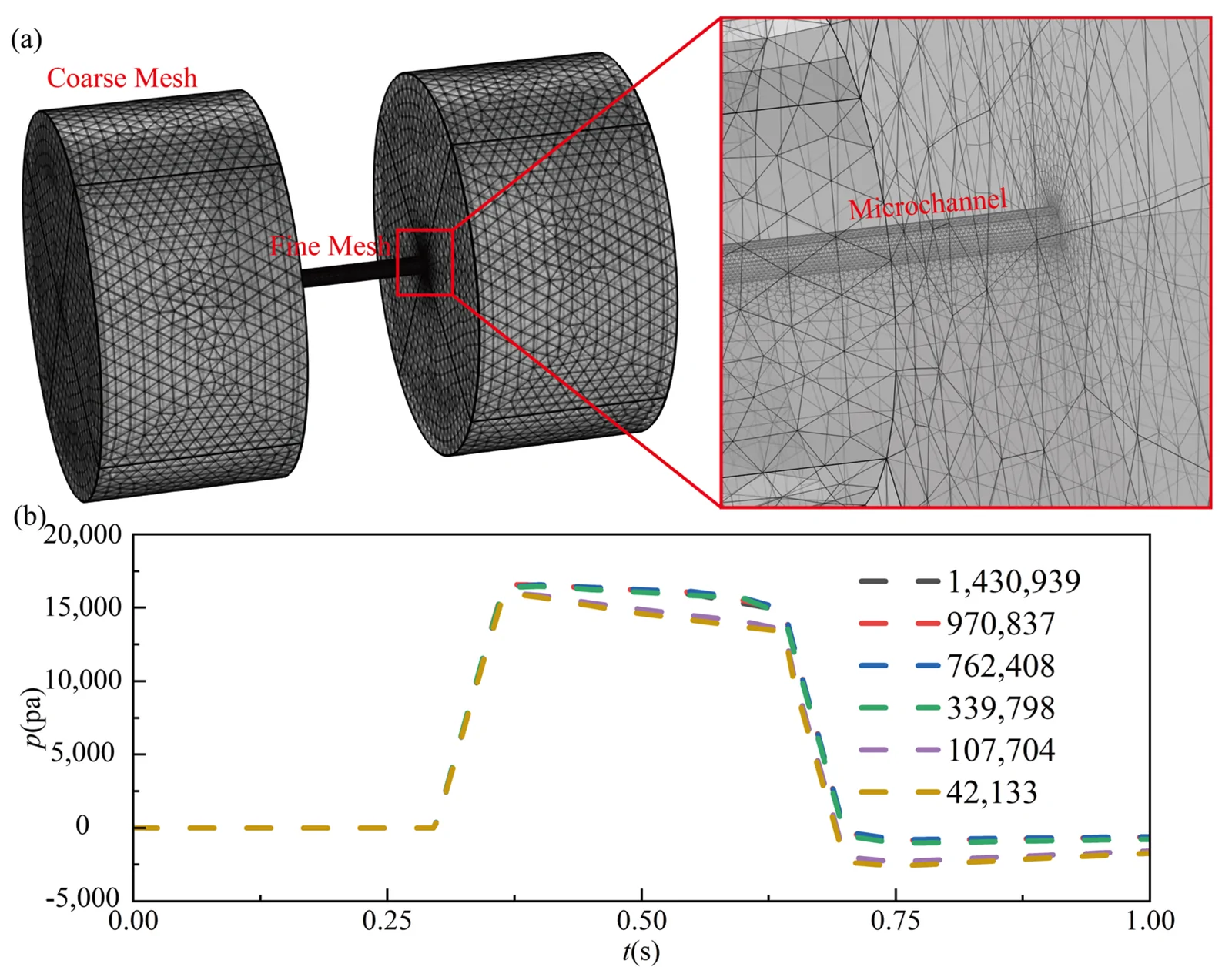
Grid-independence verification: (**a**) overall mesh configuration and refined mesh in the microchannel; (**b**) temporal evolution of the pressure in the hot reservoir under square-wave conditions for six different mesh counts.

Accurate capture of wall thermal transpiration flow and centreline Poiseuille flow in the microchannel critically depends on the mesh resolution. In this study, an unstructured meshing strategy is adopted (Figure 3a), with coarse grids applied to the macroscopic hot and cold reservoirs and refined grids concentrated in the microchannel. The maximum and minimum element sizes in the coarse region are 6.24 and 1.87 μm, respectively, with a maximum element growth rate of 1.2. Slip-flow numerical simulations are performed using the commercial software COMSOL Multiphysics 6.3, where the slip boundary condition is imposed on the microchannel walls and the no-slip [20] condition on the reservoir walls. The microchannel is thermally insulated during energy exchange. The physics-controlled settings govern the convergence tolerance, and the threshold for updating the residual ratio is set to 100. The PARDISO solver [31,32] is employed for transient algebraic equation solving, with the linear solver set to direct and a fully coupled approach for the fluid-flow variables.

For transient simulations of the hydrogen Knudsen compressor, mesh independence verification does not rely on global refinement but rather on the resolution of the Knudsen layer adjacent to the wall and the coupling between the time step and spatial scale. Accordingly, we directly vary the maximum and minimum mesh counts in the microchannel, yielding six mesh numbers ranging from 42,133 to 1,430,939. Transient simulations are conducted under a square-wave temperature variation in the hot reservoir with a time interval of 0.025 s. Figure 3b presents the temporal evolution of the temperature at the centre of the hot reservoir. When the mesh count exceeds 330,000, the error in the pressure temporal variation remains within 2%. Considering the need for refined analysis of the velocity distribution in the microchannel and the trade-off between computational cost and time, we select an unstructured mesh of 970,000 elements, with maximum and minimum element sizes of 0.027 and 0.01 μm, respectively.

**Figure 4:**
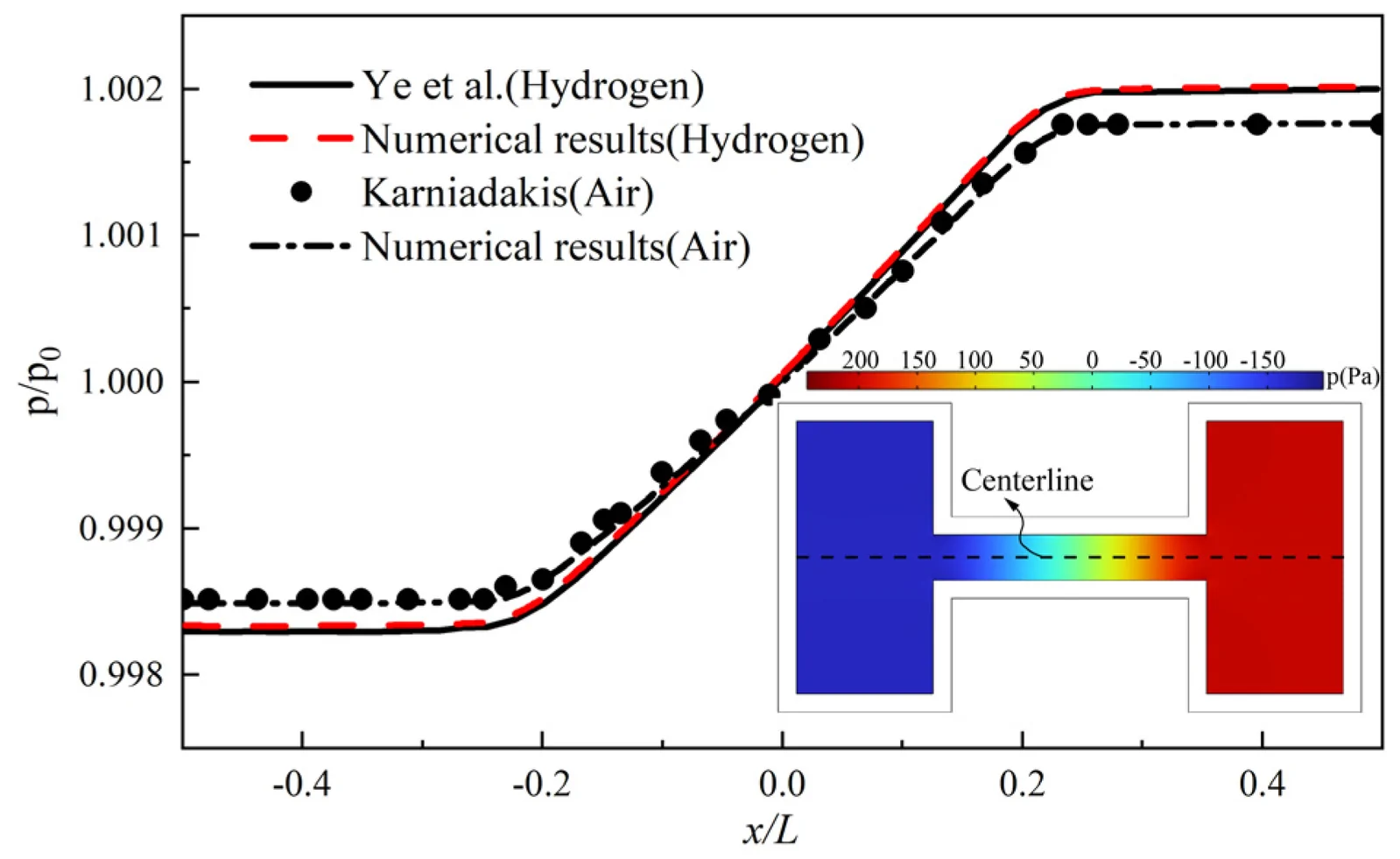
Periodic-flow vector fields for the piecewise wave, Ye et al. [1].

The computational model was rigorously validated against the benchmark case of a Knudsen compressor reported by Ye et al. [1] and Karniadakis et al. [33]. Figure 4 presents the streamwise distribution of the normalized pressure along the microchannel centreline, with a direct comparison between the present results and the reference data. The two datasets exhibit consistent trends throughout the entire flow domain. In the near-wall region, where the pressure gradient is steepest, the maximum relative deviation is within 0.3%. This high level of quantitative agreement not only confirms the validity of the computational grid used, but also further verifies the correctness of the adopted thermophysical properties and slip-boundary treatment, thereby providing a reliable numerical foundation for subsequent analyses of transient operating conditions.

## 3. Analysis of Temperature Evolution Waveforms

A systematic analysis of pressure evolution at the high-pressure and low-pressure points at both ends of the circular microchannel, combined with the velocity response along the central axis, reveals common patterns in gas flow behaviour under six periodic temperature loading waveforms (rectangular, piecewise, square, Gaussian pulse, triangular, and sinusoidal).

### 3.1. Common Regularities in Thermal Non–Equilibrium Processes

#### 3.1.1. Reversal of Pressure Gradient and Alternation of Dominant Flow Modes

**Figure 5:**
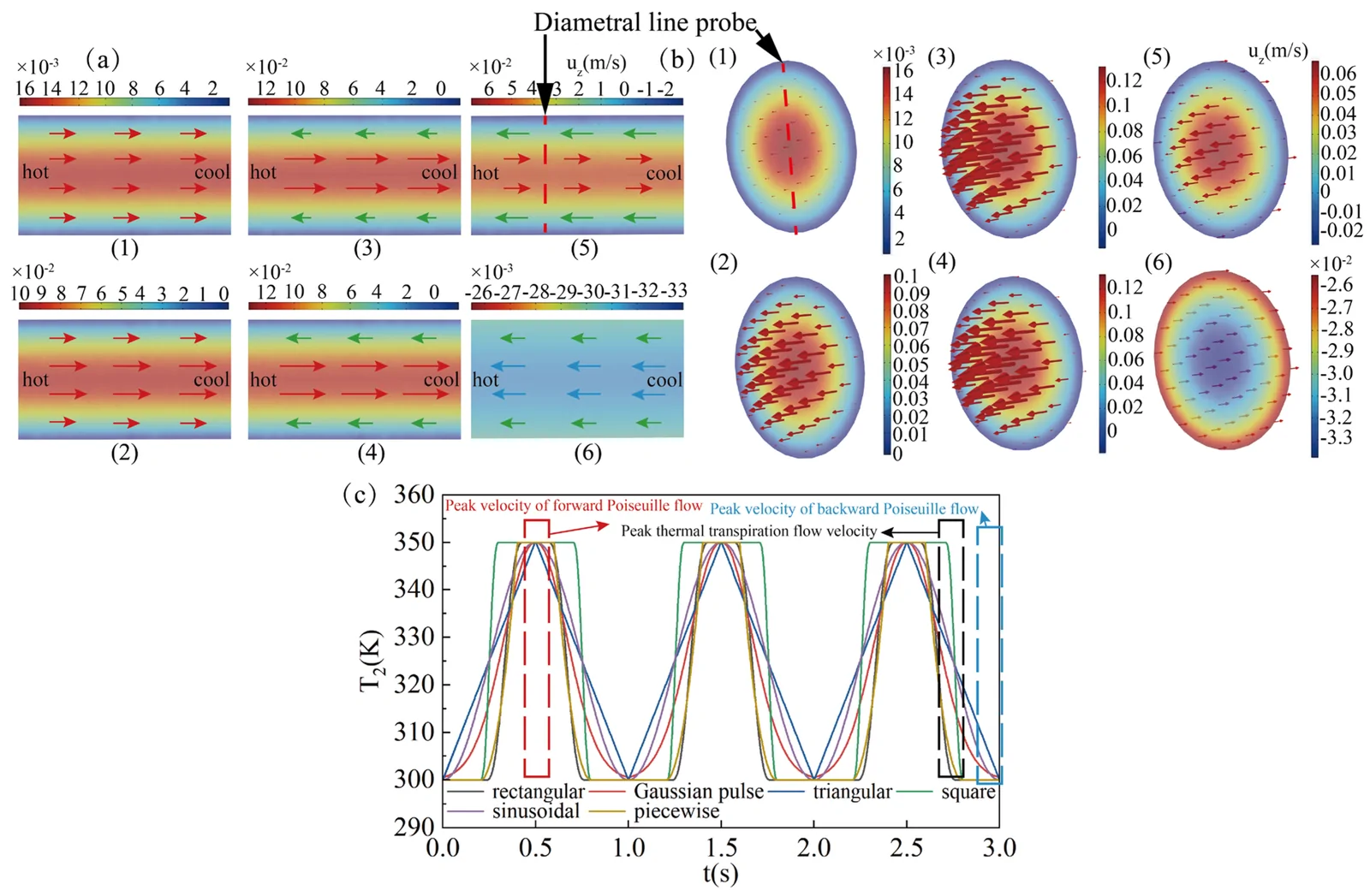
The pressure gradient and alternation of dominant flow modes: (**a**) Two-dimensional flow vector fields in the microchannel at different stages; (**b**) three-dimensional flow vector fields at the microchannel centreline at different stages; (**c**) cyclic distribution of peak velocities of the flow states.

During each heating phase, as the hot-end temperature rises from 300 K to 350 K, hydrogen in the hot reservoir expands, sharply increasing its volume. This creates a positive pressure gradient across the microchannel from the hot end to the cold end. The resulting gradient drives a Poiseuille flow in the positive z-direction, which is the main mass-transport mechanism during this stage (points (1)–(4) in Figure 5a,b). When cooling begins and the hot-end temperature drops from 350 K to 300 K, the positive pressure gradient gradually decreases as the gas cools and contracts, weakening the forward Poiseuille flow (point (5) in Figure 5a,b). At this stage, wall thermal transpiration flow, driven by the temperature gradient, surpasses the remaining forward Poiseuille flow and induces reverse hydrogen transport toward the hot end [34,35,36]. However, as the density contraction from the cooling hot end dominates, the absolute pressure in the hot reservoir continues to fall, eventually reversing the pressure gradient. This forms a negative pressure gradient from the cold end to the hot end, which then drives a Poiseuille flow in the negative z-direction, becoming the main flow pattern in the channel center during this period (point (6) in Figure 5a,b). For clarity in subsequent analysis, forward or backward Poiseuille flow refers to the axial velocity component along the microchannel centerline. Velocity in the positive z-direction (from hot to cold end) is defined as forward Poiseuille flow, while velocity in the negative z-direction (from cold to hot end) is defined as backward Poiseuille flow. Changes in the magnitude and direction of the maximum centerline velocity clearly indicate a reversal of the pressure gradient.

#### 3.1.2. Triggering Conditions and Intensity Evolution

Wall thermal transpiration flow refers to the z-axis component of the wall slip velocity, **u***_slip_*, as defined by the slip boundary condition in Equation (2). This effect appears as a macroscopic velocity slip of the gas relative to the wall at the surface. Quantifying the slip flow using **u***_slip_* accurately represents thermal transpiration and allows clear separation from the centerline Poiseuille flow in both space and time. Wall thermal transpiration flow initially appears during the heating stage of the first cycle, becoming active once a sufficiently pronounced temperature gradient is established within the microchannel, and continues throughout subsequent cycles (as shown at point (3) in Figure 5a,b). In the high-temperature regime, where the hot-end temperature approaches 350 K and the temperature gradient remains stable and steep. The thermal transpiration velocity demonstrates sustained growth, indicating strong reverse transport capacity (points (4) and (5) in Figure 5a,b). When the hot-end temperature decreases to the low-temperature range near the cold-end temperature of 300 K, the temperature gradient is substantially weakened or may temporarily disappear, resulting in a corresponding decay in thermal transpiration velocity (point (6) in Figure 5a,b). These observations indicate that the presence and intensity of thermal transpiration flow are directly dependent on the magnitude of the temperature gradient along the channel, demonstrating pronounced transient sensitivity.

#### 3.1.3. Modulation of the Three Flow Component Evolutions

As the cycle number increases, the forward Poiseuille flow velocity at a given phase in each cycle gradually decreases, while the velocities of the backward Poiseuille flow and wall thermal transpiration flow continuously increase. The enhancement of thermal transpiration flow results from the progressive establishment of a quasi-steady-state temperature-gradient distribution over multiple cycles, which increases the efficiency of thermal transpiration during each heating phase. The cycle-to-cycle variation in Poiseuille flow is attributed to the disruption of the pressure gradient by the thermal transpiration flow. This disruption leads to a reduced positive pressure difference that can be re-established in subsequent heating phases and an increased negative pressure difference during cooling phases. Therefore, the forward Poiseuille flow velocity decreases with each cycle, whereas the backward Poiseuille flow velocity increases. As illustrated in Figure 5c, across all waveform conditions, the maximum forward Poiseuille flow velocity occurs near the first heating peak (time window around 0.4–0.5 s), the maximum backward Poiseuille flow velocity appears near the last cooling valley (approximately 2.8–3.0 s), and the maximum wall thermal transpiration flow velocity is observed slightly before the peak of the backward Poiseuille flow (around 2.7–2.8 s). These observations reflect the temporal sequence and competitive interactions among the three flow components.

#### 3.1.4. Suppression of Poiseuille Flow Development

In temperature waveforms, the presence of an isothermal hold time (plateau segment) at either high temperature (350 K) or low temperature (300 K) exerts opposing effects on Poiseuille flow and thermal transpiration flow. An extended isothermal hold time increasingly impedes the full development of Poiseuille flow, as the flow velocity within the channel decreases with prolonged hold time. The underlying physical mechanism is that, during the isothermal stage, although the temperature gradient disappears, wall-driven thermal transpiration flow persists. If a density inhomogeneity remains across the channel ends due to previous temperature differences, the thermal transpiration effect continuously transports hydrogen in the reverse direction, from the cold end to the hot end, gradually counteracting and disrupting the original pressure gradient. Conversely, a high-temperature isothermal region supports the sustained maintenance of the temperature gradient, thereby promoting the establishment and enhancement of thermal transpiration flow. In contrast, a low-temperature isothermal region eliminates the temperature gradient and inhibits the development of thermal transpiration flow. This behavior is consistently observed in all waveforms containing plateau segments, such as rectangular and square waves.

### 3.2. Waveform Specific Analysis of Temperature Evolution

#### 3.2.1. Rectangular Wave

**Figure 6:**
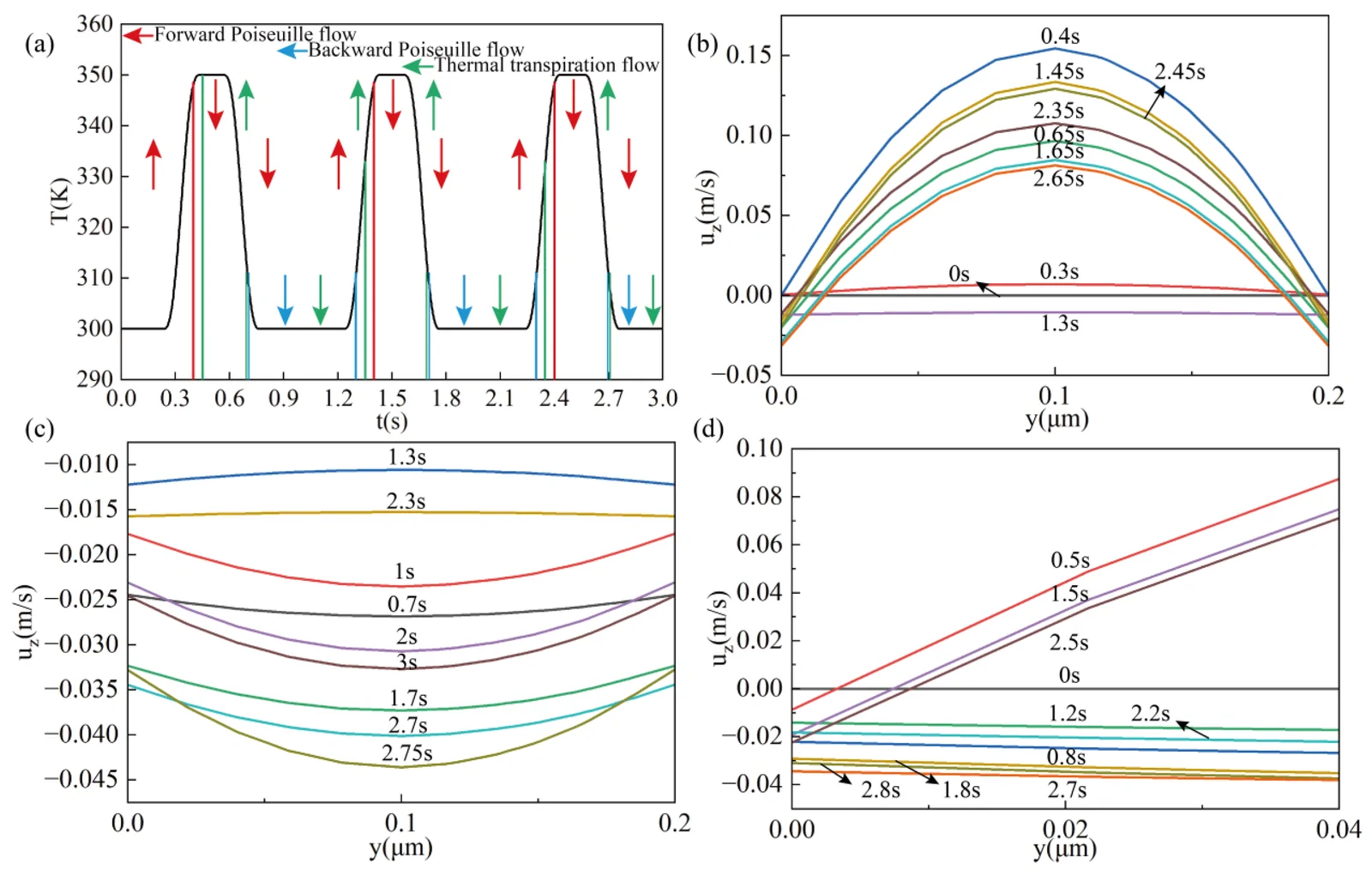
Temporal and spatial distributions of physical variables under rectangular wave conditions: (**a**) Rectangular waveform; (**b**) forward Poiseuille flow velocity distribution (0.3 s–0.65 s, 1.35 s–1.65 s, 2.35 s–2.65 s); (**c**) backward Poiseuille flow velocity distribution (0.7 s–1.3 s, 1.7 s–2.3 s. 2.7 s–3.0 s); (**d**) thermal transpiration flow velocity distribution (0.45 s–3 s).

The velocities of thermal transpiration flow and Poiseuille flow along the diametral sampling line of the circular microchannel cross-section. These velocities represent the axial components along the microchannel axis. Figure 6a demonstrates that the rectangular-wave temperature loading produces instantaneous step changes in the hot-end temperature between 300 K and 350 K, maintaining constant plateaus at both extremes. The forward Poiseuille flow at the center of the circular microchannel is present during 0.3–0.65 s, 1.35–1.65 s, and 2.35–2.65 s, with effective development intervals of 0.3–0.4 s, 1.35–1.4 s, and 2.35–2.4 s, respectively. Figure 6b indicates that the peak velocity of the forward Poiseuille flow occurs near the first heating peak at 0.4 s, reaching 0.1545 m/s. The backward Poiseuille flow at the microchannel center is observed during the low-temperature intervals of 0.7–1.3 s, 1.7–2.3 s, and 2.7–3.0 s, with effective development periods of 0.7–0.75 s, 1.7–1.75 s, and 2.7–2.75 s. According to Figure 6c, the peak of the backward Poiseuille flow, reaching −0.0436 m/s, occurs at the onset of the low-temperature period in the final cycle (2.75 s). The wall thermal transpiration flow persists from its initiation at 0.45 s, with effective development during 0.45–0.7 s, 1.35–1.7 s, and 2.35–2.7 s, and weakens during 0.7–1.35 s, 1.7–2.35 s, and 2.7–3.0 s. Figure 6d shows that the peak value of this flow appears at 2.7 s, reaching −0.0344 m/s.

**Figure 7:**
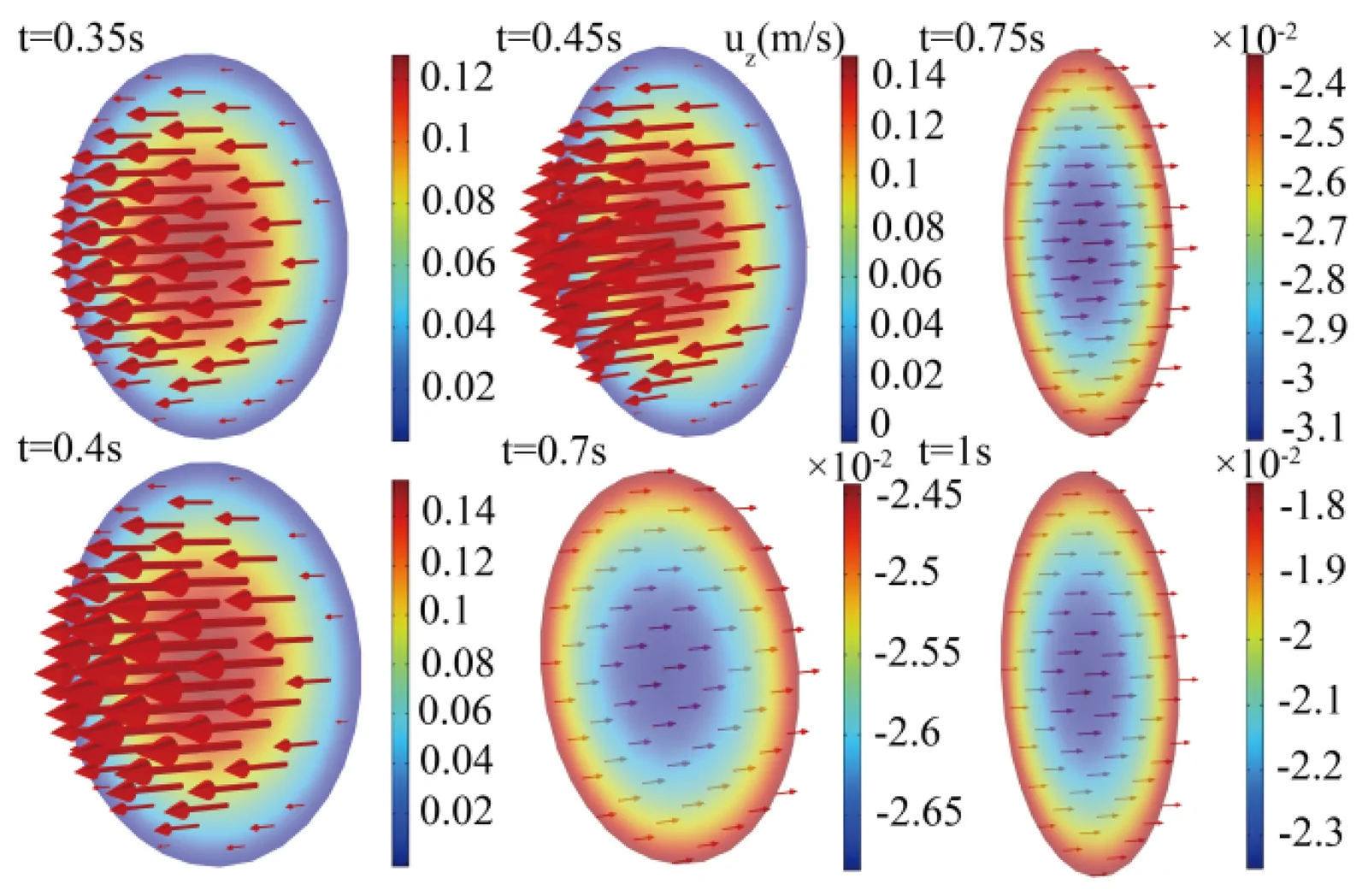
Periodic-flow vector fields for the rectangular wave.

Figure 7 depicts the evolution of flow during the first cycle under rectangular-wave loading, as indicated by the flow vector field. At the initial heating stage (*t* = 0.35 s), rapid heating and expansion of the gas in the hot reservoir establish a positive pressure gradient across the circular tube from the hot end to the cold end. This gradient generates a parabolic velocity profile in the central region, demonstrating the predominance of forward Poiseuille flow. As the hot-end temperature rapidly increases to 350 K and is maintained, the pressure difference between the hot and cold ends continues to rise, further intensifying the central forward Poiseuille flow. The maximum velocity occurs at *t* = 0.4 s, corresponding to the peak pressure difference. During the subsequent high-temperature isothermal plateau, the forward Poiseuille flow velocity decreases as the pressure difference gradually lessens due to ongoing gas transport from the hot end to the cold end. By *t* = 0.45 s, the high-temperature plateau has persisted long enough for the temperature gradient to stabilize, resulting in the onset of a negative wall thermal transpiration flow. At *t* = 0.7 s, the hot end enters the cooling phase, and the pressure at the cold end surpasses that at the hot end, creating a reverse pressure gradient that induces a backward Poiseuille flow in the central region. Simultaneously, the cooling process reduces the temperature gradient, and the thermal transpiration flow reaches its cycle peak before its velocity gradually declines. During the low-temperature isothermal plateau (*t* = 0.75 s to 1.0 s), the thermal transpiration flow persists, continuously transferring gas from the cold end back to the hot end. This process disrupts the reverse pressure gradient and causes the backward Poiseuille flow to diminish steadily. As the temperatures of the hot and cold ends equalize, the temperature gradient vanishes, resulting in the decay of the thermal transpiration flow.

#### 3.2.2. Piecewise Wave

**Figure 8:**
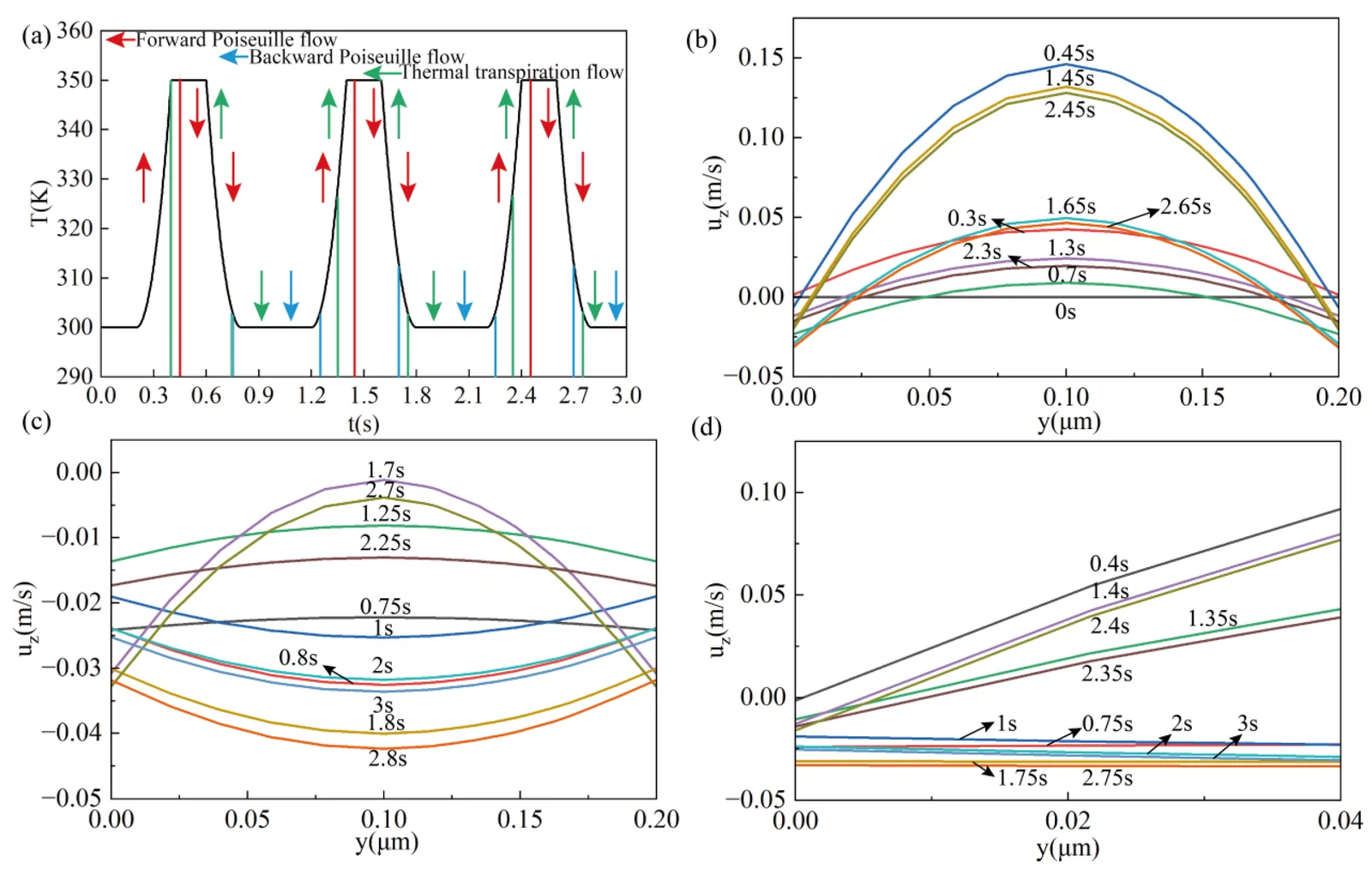
Temporal and spatial distributions of physical variables under piecewise wave conditions: (**a**) piecewise waveform; (**b**) forward Poiseuille flow velocity distribution (0.2 s–0.7 s, 1.3 s–1.65 s, 2.3–2.65 s); (**c**) backward Poiseuille flow velocity distribution (0.75 s–1.25 s, 1.7 s–2.25 s, 2.7 s–3.0 s); (**d**) thermal transpiration flow velocity distribution (0.45 s–3 s).

The piecewise waveform exhibits parabolic heating and cooling curves with progressively increasing slopes (Figure 8a), as well as constant plateaus in both high- and low-temperature regions. The flow regime distribution in Figure 8a indicates that forward Poiseuille flow occurs during 0.2–0.7 s, 1.3–1.65 s, and 2.3–2.65 s, with effective development durations of 0.2–0.45 s, 1.3–1.45 s, and 2.3–2.45 s.

According to the cycle-to-cycle variation in the forward Poiseuille flow velocity shown in Figure 8b, the peak value is observed at 0.45 s, reaching 0.1462 m/s. The backward Poiseuille flow is present during the low-temperature plateau periods of 0.75–1.25 s, 1.7–2.25 s, and 2.7–3.0 s, with effective development intervals of 0.75–0.8 s, 1.7–1.8 s, and 2.7–3.0 s. Figure 8c shows that the peak of the backward Poiseuille flow reaches −0.0424 m/s at 2.8 s. The wall thermal transpiration flow persists from its initiation at 0.4 s, with effective development during 0.4–0.75 s, 1.35–1.75 s, and 2.35–2.75 s. Its peak velocity, as shown in Figure 8d, is −0.033 m/s at 2.75 s.

**Figure 9:**
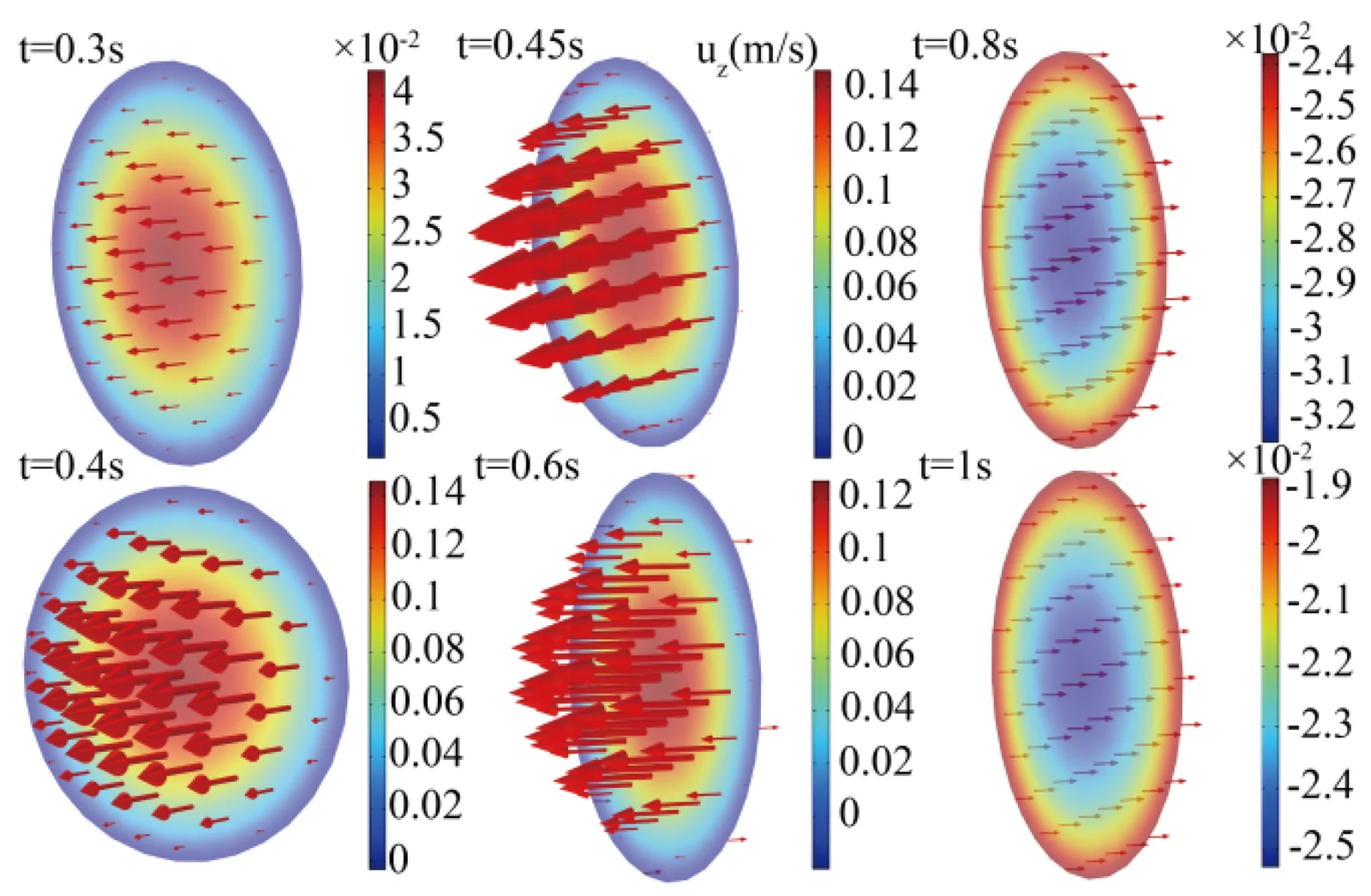
Periodic-flow vector fields for the piecewise wave.

Figure 9 presents the evolution of flow during the first cycle under piecewise waveform loading, as indicated by the flow vector field. At the initial heating stage (*t* = 0.3 s), gas expansion in the hot reservoir generates a positive pressure gradient from the hot end to the cold end. This gradient produces a parabolic velocity profile at the center of the circular tube, with forward Poiseuille flow dominating. As the hot-end temperature rises, the pressure difference between the two ends increases, further intensifying the central forward Poiseuille flow. At *t* = 0.4 s, the hot-end temperature reaches 350 K and enters the high-temperature isothermal region. Here, the temperature gradient becomes more pronounced, and a negative thermal transpiration flow develops near the wall. Around *t* = 0.45 s, the pressure difference reaches its maximum, and the forward Poiseuille flow velocity at the center attains its peak for the cycle. During the high-temperature hold period, the wall thermal transpiration flow continues to increase and gradually extends toward the tube’s central region. This reverse transport effect reduces the positive pressure gradient, resulting in a decrease in forward Poiseuille flow velocity. After *t* = 0.6 s, cooling at the hot end further diminishes the forward Poiseuille flow. By *t* = 0.75 s, the cold-end pressure exceeds that of the hot end, establishing a reverse pressure gradient and initiating backward Poiseuille flow in the central region. Simultaneously, the cooling process weakens the temperature gradient, and the thermal transpiration flow reaches its cycle maximum before its velocity decreases. At *t* = 0.8 s, the reverse pressure difference increases, further strengthening the backward Poiseuille flow. Its velocity surpasses that of the wall thermal transpiration flow, resulting in a parabolic velocity distribution across the cross-section directed from the cold end to the hot end, with the backward Poiseuille flow reaching its cycle maximum. From *t* = 0.8 s to 1.0 s, during the low-temperature isothermal period and the final cooling stage, the temperature reaches its minimum, and both the backward Poiseuille flow and the thermal transpiration flow decrease.

#### 3.2.3. Square Wave

**Figure 10:**
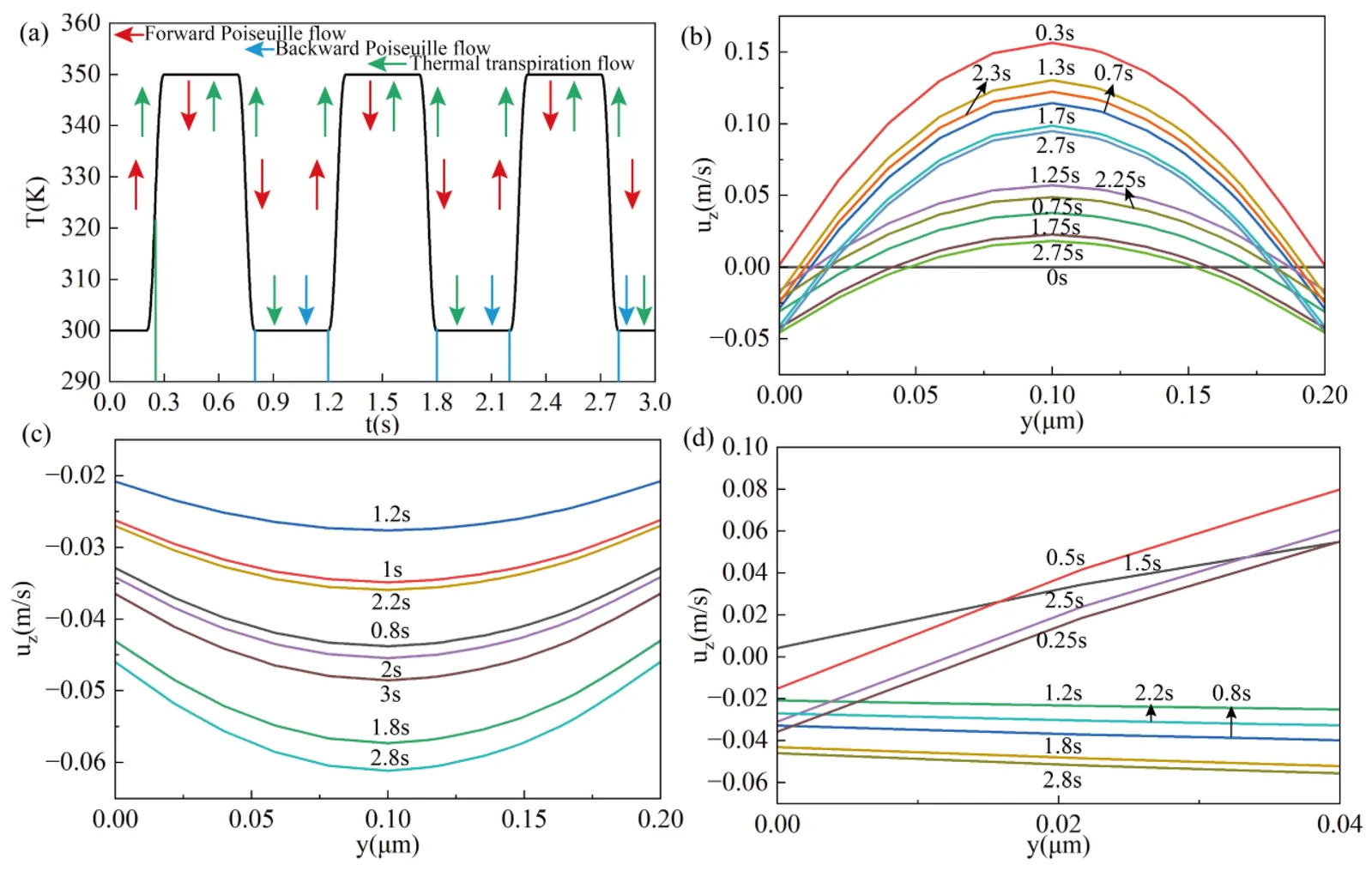
Temporal and spatial distributions of physical variables under square-wave conditions: (**a**) piecewise waveform; (**b**) forward Poiseuille flow velocity distribution (0.25 s–0.75 s, 1.25 s–1.75 s, 2.25 s–2.75 s); (**c**) backward Poiseuille flow velocity distribution (0.8 s–1.2 s, 1.8 s–2.2 s, 2.8 s–3.0 s); (**d**) thermal transpiration flow velocity distribution (0.45 s–3 s).

Figure 10a illustrates that the square wave represents an extreme waveform characterized by instantaneous step changes and distinct isothermal plateaus, accompanied by exceptionally high heating and cooling rates and minimal transition durations. The flow regimes in Figure 10a indicate that forward Poiseuille flow occurs during 0.25–0.75 s, 1.25–1.75 s, and 2.25–2.75 s, with effective development intervals at 0.2–0.3 s, 1.2–1.3 s, and 2.2–2.3 s. According to Figure 10b, the peak forward Poiseuille flow velocity is observed at 0.3 s, reaching 0.1566 m/s. Backward Poiseuille flow is present during the low-temperature plateau periods of 0.8–1.2 s, 1.8–2.2 s, and 2.8–3.0 s. Figure 10c shows that the peak backward Poiseuille flow velocity occurs at 2.8 s, with a value of −0.0611 m/s. Wall thermal transpiration flow persists from its onset at 0.25 s, with effective development during 0.25–0.8 s, 1.2–1.8 s, and 2.2–2.8 s. As depicted in Figure 10d, the peak wall thermal transpiration flow velocity is recorded at 2.8 s, with a magnitude of −0.046 m/s. The peak velocities of all three flow components for the square wave are the highest among the six waveforms examined. Due to the extended residence time in the high-temperature region, the thermal transpiration flow intensity for the square wave substantially exceeds that of the other waveforms. Furthermore, the shortest heating and cooling durations and the highest rates result in the largest forward and backward Poiseuille flow intensities.

**Figure 11:**
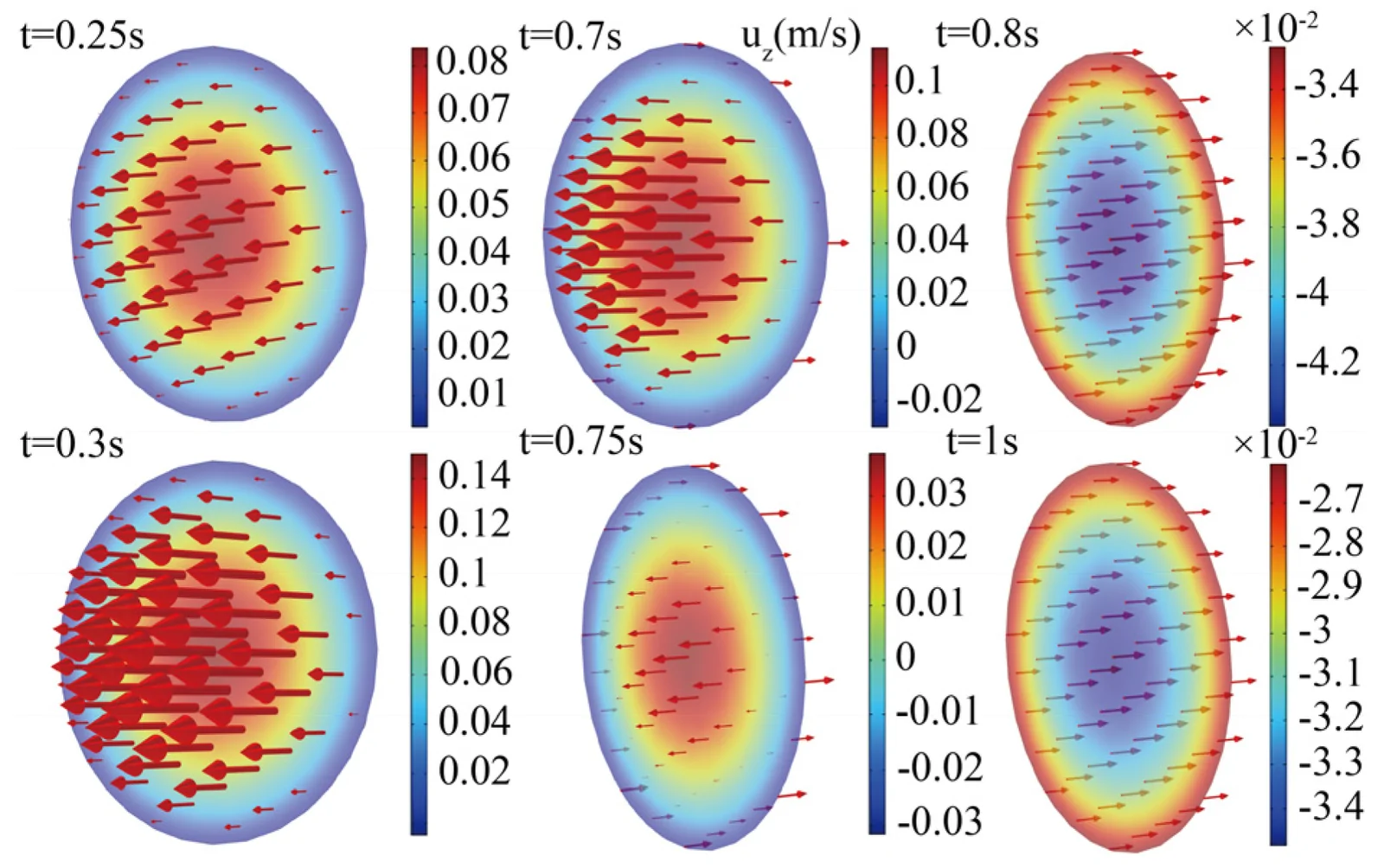
Periodic-flow vector fields for the square wave.

Figure 11 illustrates the flow evolution process during the first cycle under square-wave loading, as revealed by the flow vector field. At the initial heating stage (*t* = 0.25 s), rapid heating and expansion of the gas in the hot reservoir establish a strong positive pressure gradient across the circular tube. This gradient drives a parabolic velocity profile in the central region, resulting in the rapid dominance of forward Poiseuille flow. As the hot-end temperature remains elevated, the pressure difference between the hot and cold ends increases, further strengthening the central forward Poiseuille flow. The rapid formation of the temperature gradient leads to the early appearance of a negative thermal transpiration flow in the near-wall region. At *t* = 0.3 s, when the hot-end temperature and the pressure difference reach their respective maxima, the forward Poiseuille flow velocity at the centre attains its cycle peak. During the subsequent high-temperature isothermal plateau, the wall thermal transpiration flow continues to intensify and gradually extends toward the tube centre. This reverse mass-transport effect reduces the pressure difference between the hot and cold ends, causing a corresponding weakening of the forward Poiseuille flow. During the cooling transition (*t* = 0.7 s to 0.8 s), the hot-end temperature decreases, and the pressure difference continues to decline, further diminishing the forward Poiseuille flow. Meanwhile, the wall thermal transpiration flow strengthens due to the brief persistence of the temperature gradient. At *t* = 0.8 s, the hot end enters the low-temperature isothermal plateau. The cold-end pressure surpasses that of the hot end, establishing a reverse pressure gradient from the cold end to the hot end, and the backward Poiseuille flow in the central region reaches its peak instantaneously. Simultaneously, the thermal transpiration flow also attains its cycle maximum. During the subsequent low-temperature hold period (*t* = 0.8 s–1.0 s), both the backward Poiseuille flow and the thermal transpiration flow decay as pressure equilibrium is gradually restored and the temperature gradient dissipates.

#### 3.2.4. Gaussian Pulse

**Figure 12:**
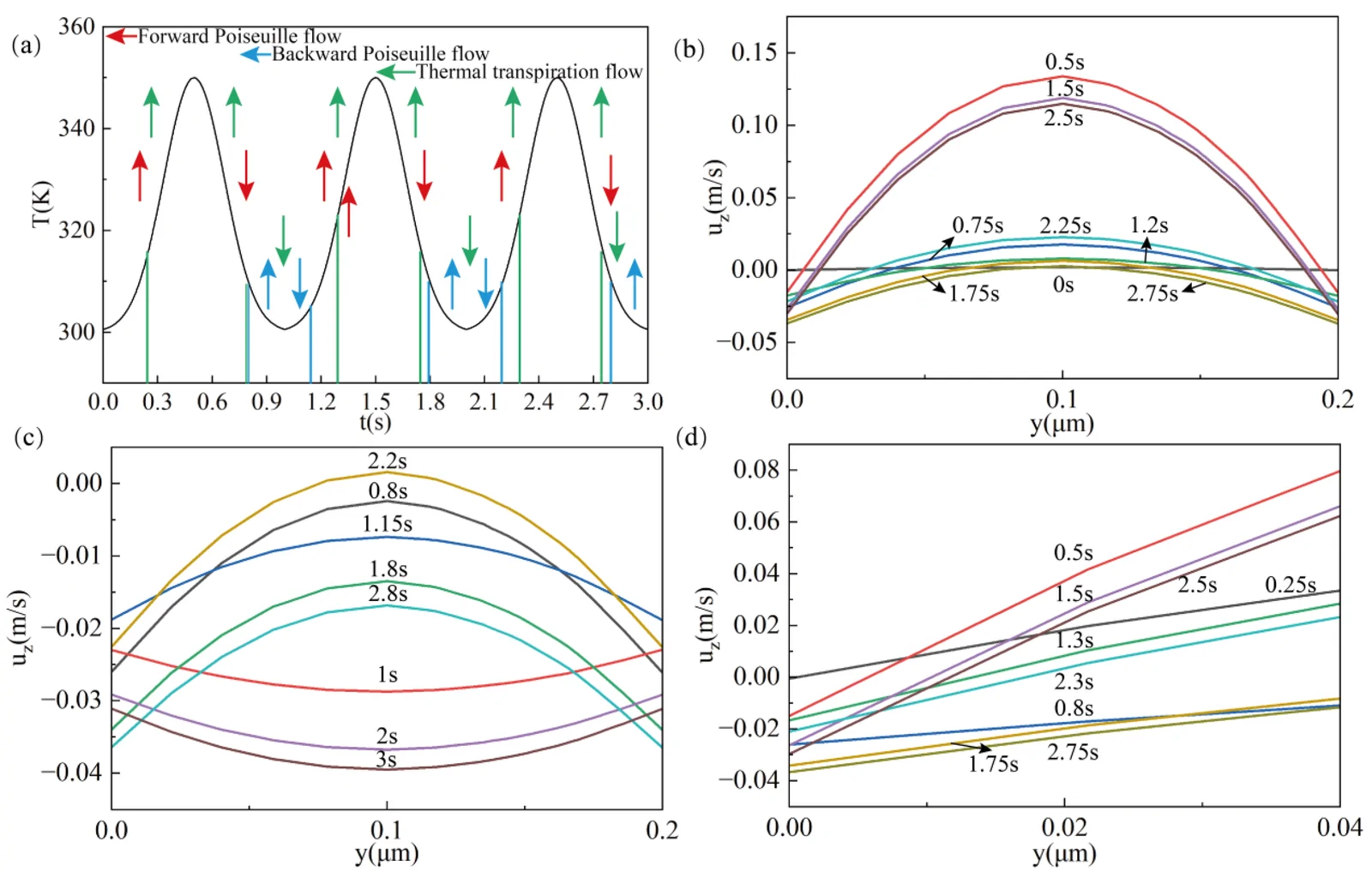
Temporal and spatial distributions of physical variables under Gaussian pulse conditions: (**a**) piecewise waveform; (**b**) forward Poiseuille flow velocity distribution (0 s–0.75 s, 1.2 s–1.75 s, 2.25 s–2.75 s); (**c**) backward Poiseuille flow velocity distribution (0.8 s–1.15 s,1.8 s–2.2 s,2.8 s–3.0 s); (**d**) thermal transpiration flow velocity distribution (0.45 s–3 s).

Figure 12a illustrates that the Gaussian pulse produces a smooth, bell-shaped temperature curve without isothermal plateaus. According to the flow regime distribution in Figure 12a, forward Poiseuille flow is observed during 0–0.75 s, 1.2–1.75 s, and 2.25–2.75 s, with effective development intervals of 0–0.5 s, 1–1.5 s, and 2–2.5 s. Figure 12b indicates that the peak forward Poiseuille flow velocity occurs at 0.5 s, reaching 0.1339 m/s. Backward Poiseuille flow is present during the low-temperature periods of 0.8–1.15 s, 1.8–2.2 s, and 2.8–3.0 s, with effective development intervals of 0.8–1 s, 1.8–2 s, and 2.8–3 s. As shown in Figure 12c, the peak backward Poiseuille flow velocity is −0.0395 m/s at 3.0 s. Wall thermal transpiration flow persists from its initiation at 0.25 s, with effective development during 0.25–0.8 s, 1.3–1.75 s, and 2.3–2.75 s. Figure 12d shows that the peak thermal transpiration flow velocity is −0.0367 m/s at 2.75 s.

**Figure 13:**
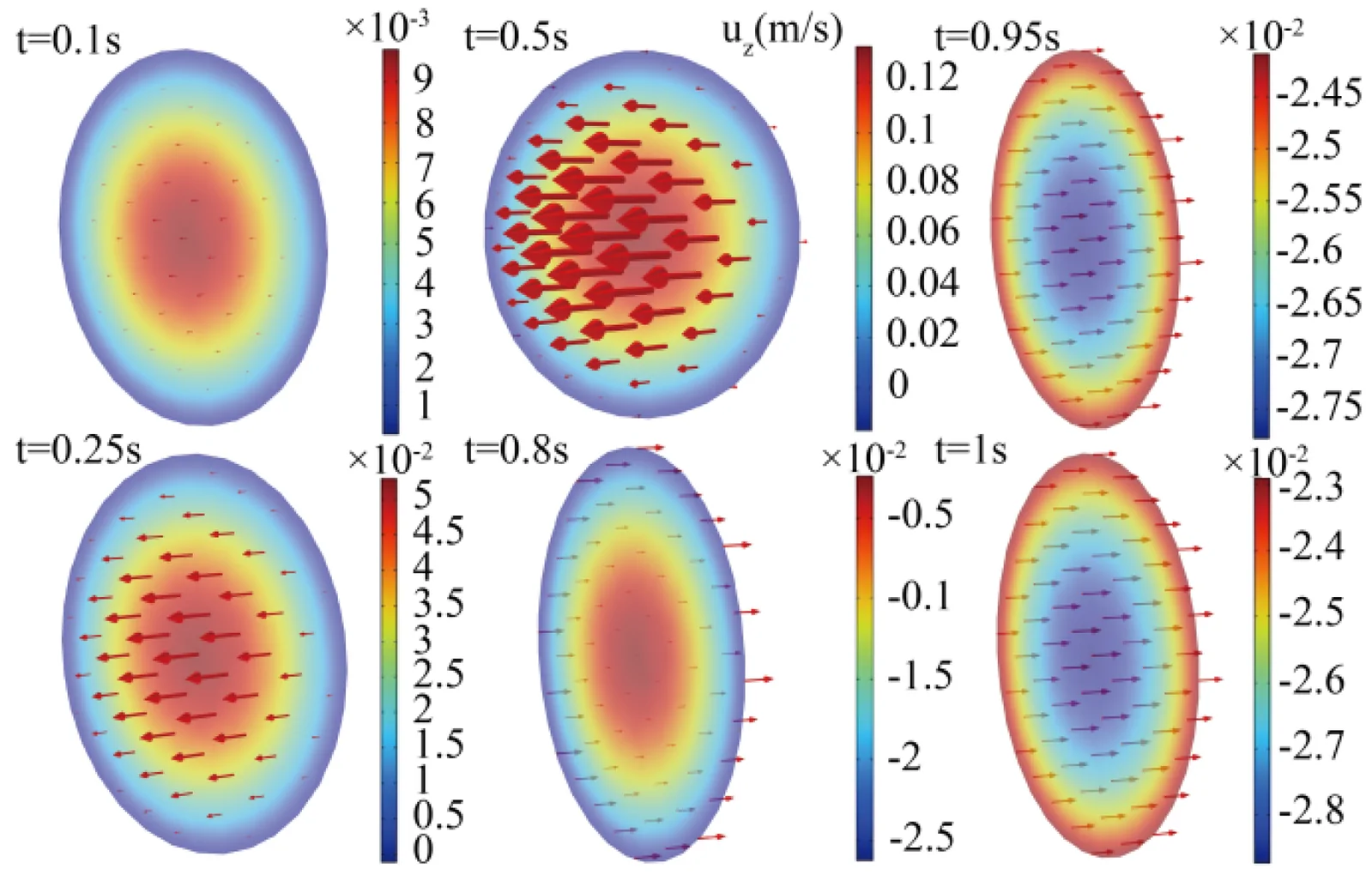
Periodic-flow vector fields for the Gaussian pulse.

Figure 13 illustrates the flow evolution during the first cycle under Gaussian pulse loading, as revealed by the flow vector field. At the initial heating stage (*t* = 0.1 s), gas expansion in the hot reservoir generates a positive pressure gradient, resulting in a parabolic velocity profile at the centre of the circular tube, with forward Poiseuille flow prevailing. As the hot-end temperature increases along a bell-shaped curve, the pressure difference between the two ends rises continuously, intensifying the central forward Poiseuille flow. By *t* = 0.25 s, the temperature gradient becomes sufficiently pronounced, and a negative thermal transpiration flow emerges in the near-wall region. At *t* = 0.5 s, when the hot-end temperature peaks at 350 K, the pressure difference between the hot and cold ends also reaches its maximum, and the forward Poiseuille flow velocity at the centre attains its cycle peak. During the subsequent cooling phase, the forward Poiseuille flow declines as the pressure difference decreases, while the temperature gradient exhibits a hysteresis effect. The wall thermal transpiration flow continues to strengthen and gradually extends toward the central region. At *t* = 0.8 s, the cold-end pressure surpasses that of the hot end, establishing a reverse pressure gradient from the cold end to the hot end, and a backward Poiseuille flow develops in the central region. Concurrently, as the cooling process weakens the temperature gradient, the thermal transpiration flow reaches its cycle peak, after which its velocity gradually decreases.

Near *t* = 0.95 s, the reverse pressure difference increases further, and the backward Poiseuille flow intensifies, with its velocity exceeding that of the wall thermal transpiration flow. The entire cross-section then exhibits a parabolic velocity distribution directed from the cold end to the hot end. At *t* = 1.0 s, when the temperature reaches its minimum, the backward Poiseuille flow velocity attains its cycle peak. As the next heating phase begins, this backward flow progressively decays.

#### 3.2.5. Triangular Wave

**Figure 14:**
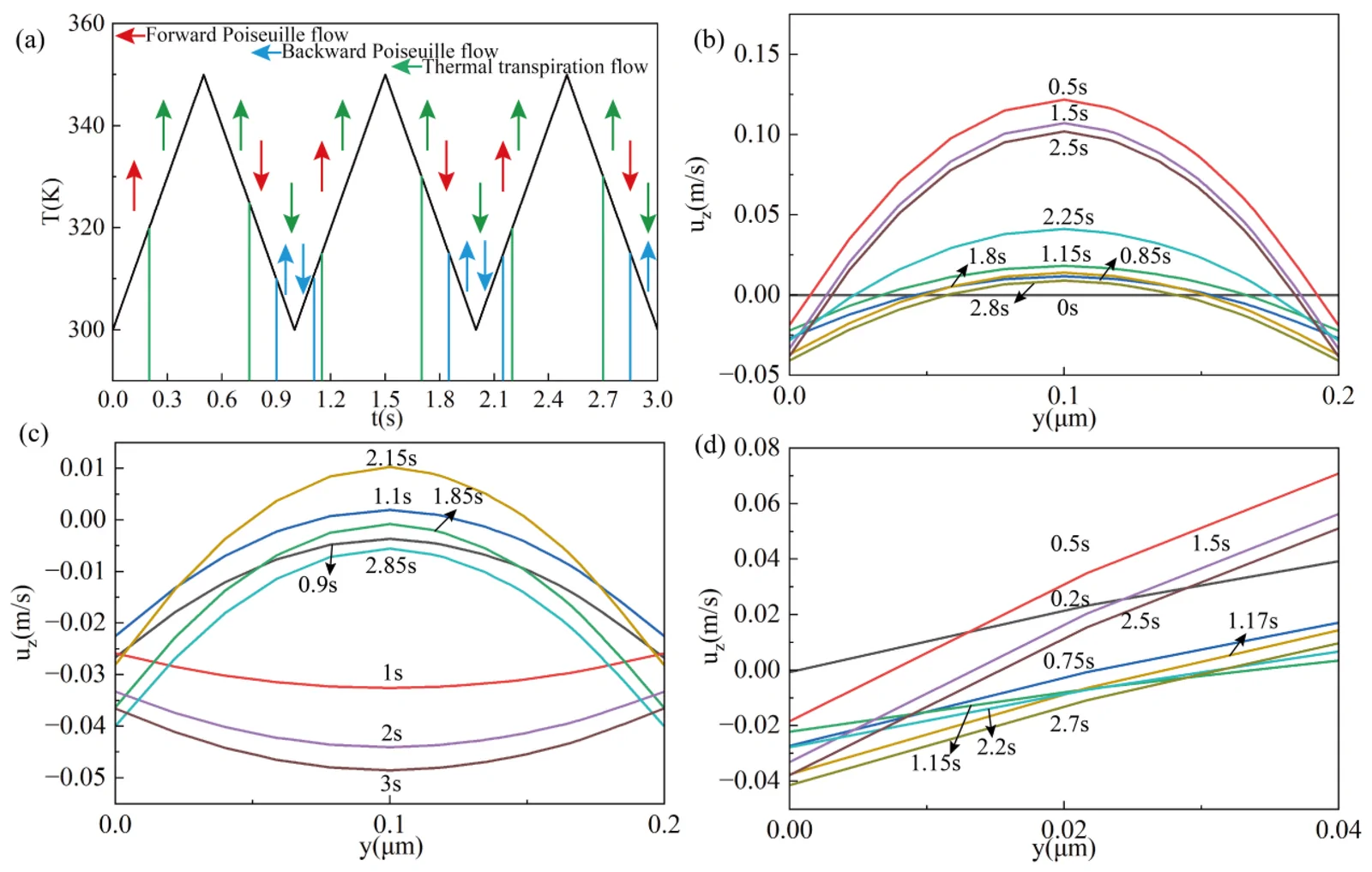
Temporal and spatial distributions of physical variables under Gaussian pulse conditions: (**a**) piecewise waveform; (**b**) forward Poiseuille flow velocity distribution (0 s–0.85 s,1.15 s–1.8 s, 2.25 s–2.8 s); (**c**) backward Poiseuille flow velocity distribution (0.9 s–1.1 s, 1.85 s–2.15 s, 2.85 s–3.0 s); (**d**) thermal transpiration flow velocity distribution (0.45 s–3 s).

Figure 14a illustrates that the triangular wave exhibits linear heating and cooling ramps, with no plateau regions. The forward Poiseuille flow is observed during 0–0.85 s, 1.15–1.8 s, and 2.25–2.8 s, with effective development intervals of 0–0.5 s, 1–1.5 s, and 2–2.5 s. According to Figure 14b, the peak forward Poiseuille flow velocity is 0.1218 m/s at 0.5 s. The backward Poiseuille flow occurs during the low-temperature periods of 0.9–1.1 s, 1.85–2.15 s, and 2.85–3.0 s, with effective development intervals of 0.9–1 s, 1.85–2 s, and 2.85–3 s. Figure 14c shows that the peak backward Poiseuille flow velocity is −0.0485 m/s at 3.0 s. The wall thermal transpiration flow persists from its initiation at 0.2 s, with effective development during 0.2–0.75 s, 1.15–1.7 s, and 2.2–2.7 s. Figure 14d indicates that the peak thermal transpiration flow velocity is −0.0414 m/s at 2.7 s.

**Figure 15:**
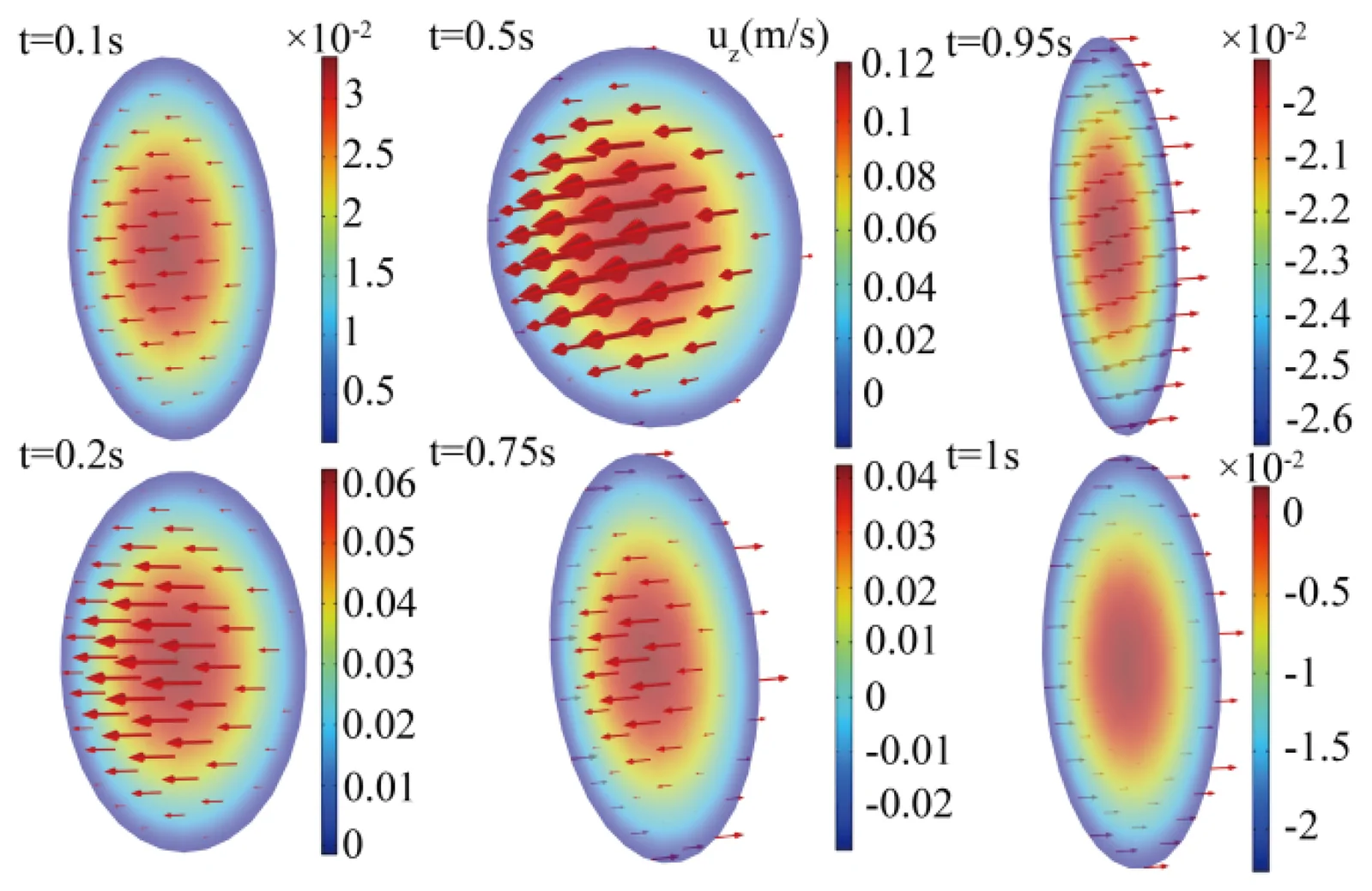
Periodic-flow vector fields for the triangular wave.

Under triangular-wave loading, the evolution of flow during the first cycle, as depicted by the flow vector field, is shown in Figure 15. At the initial heating stage (*t* = 0.1 s), gas in the hot reservoir expands as temperature increases linearly, generating a positive pressure gradient from the hot end to the cold end. This gradient drives a parabolic velocity profile at the center of the circular tube, with forward Poiseuille flow prevailing. As the hot-end temperature rises at a constant rate, the pressure difference between the two ends increases linearly, and the central forward Poiseuille flow intensifies. By *t* = 0.2 s, a temperature gradient develops, and a negative thermal transpiration flow emerges in the near-wall region. At *t* = 0.5 s, when the hot-end temperature reaches 350 K, the pressure difference between the hot and cold ends reaches its maximum, and the forward Poiseuille flow velocity at the center attains its cycle peak. During the subsequent linear cooling phase, the wall thermal transpiration flow continues to strengthen as the temperature gradient is maintained, gradually extending toward the tube center. By *t* = 0.75 s, the cooling process has substantially weakened the temperature gradient. The thermal transpiration flow reaches its cycle peak at this point, after which its velocity decreases. Near *t* = 0.95 s, the cold-end pressure exceeds that of the hot end, establishing a reverse pressure gradient from the cold end to the hot end, and a backward Poiseuille flow emerges in the central region. As the temperature continues to decrease, the backward Poiseuille flow further intensifies. At *t* = 1.0 s, when the temperature reaches its minimum of 300 K, the backward Poiseuille flow velocity attains its cycle peak, surpassing the wall thermal transpiration flow. The entire cross-section then exhibits a parabolic velocity distribution directed from the cold end to the hot end. As the next heating phase begins, this backward flow progressively decays.

#### 3.2.6. Sinusoidal Wave

**Figure 16:**
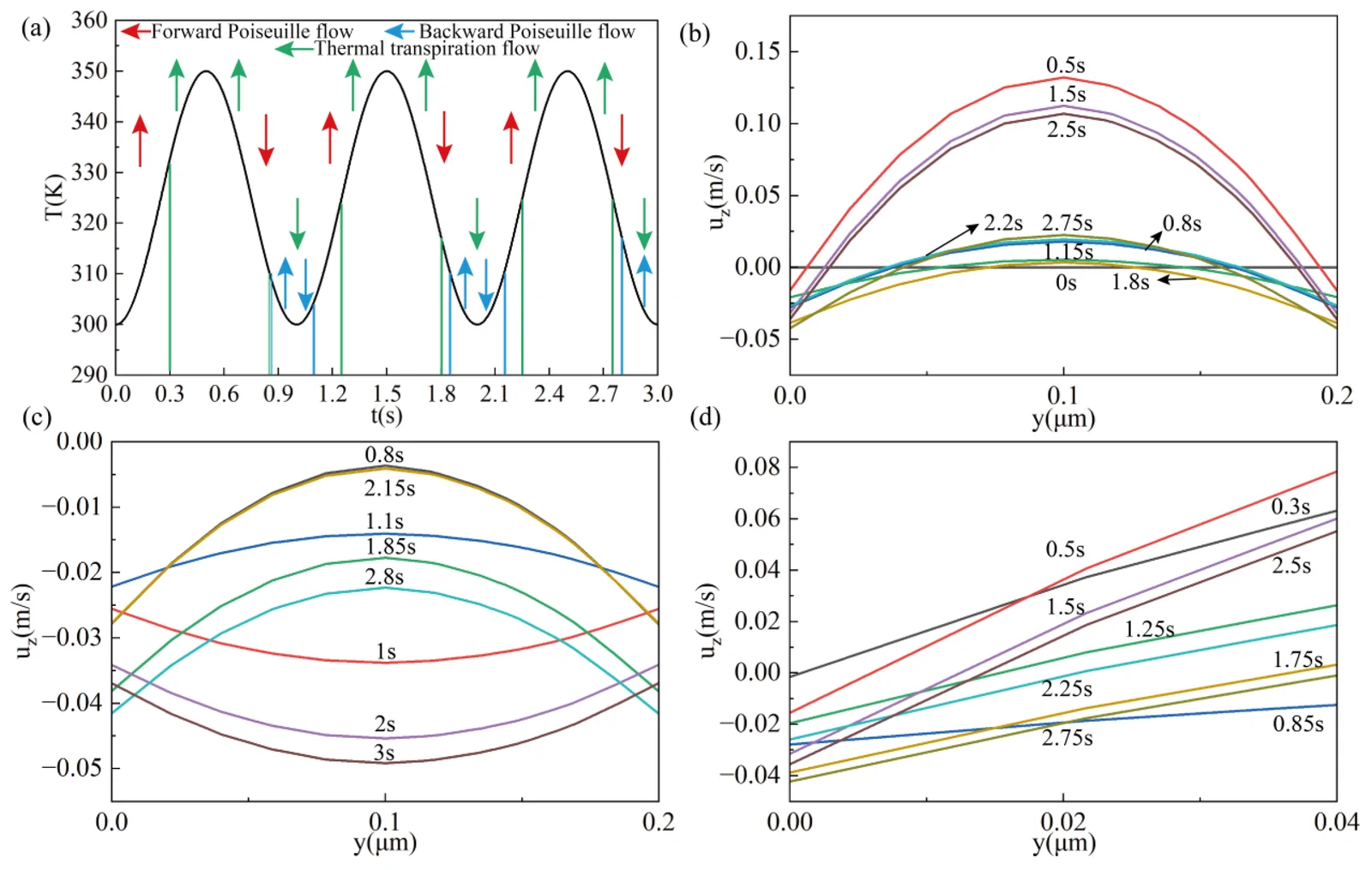
Temporal and spatial distributions of physical variables under sinusoidal wave e conditions: (**a**) piecewise waveform; (**b**) forward Poiseuille flow velocity distribution (0 s–0.85 s, 1.15 s–1.8 s, 2.25 s–2.8 s); (**c**) backward Poiseuille flow velocity distribution (0.9 s–1.1 s, 1.85 s–2.15 s, 2.85 s–3.0 s); (**d**) thermal transpiration flow velocity distribution (0.45 s–3 s).

Figure 16a illustrates that the sinusoidal waveform produces a smooth and continuous temperature variation. The heating and cooling rates reach their maximum at the midpoints of the ramps and decrease to zero at the extrema, without any plateau phases. Forward Poiseuille flow is observed during 0–0.8 s, 1.15–1.8 s, and 2.2–2.75 s, with effective development intervals of 0–0.5 s, 1–1.5 s, and 2–2.5 s. According to Figure 16b, the peak forward Poiseuille flow velocity is 0.1325 m/s at 0.5 s. Backward Poiseuille flow occurs during the low-temperature intervals of 0.85–1.1 s, 1.85–2.15 s, and 2.8–3.0 s, with effective development intervals of 0.85–1 s, 1.85–2 s, and 2.8–3 s. Figure 16c shows that the peak backward Poiseuille flow velocity is −0.0492 m/s at 3.0 s. Wall thermal transpiration flow begins at 0.3 s, develops effectively during 0.3–0.85 s, 1.25–1.75 s, and 2.25–2.75 s, and decays during 0.85–1.25 s, 1.75–2.25 s, and 2.75–3.0 s. As indicated in Figure 16d, the peak thermal transpiration flow velocity is −0.0423 m/s at 2.75 s.

**Figure 17:**
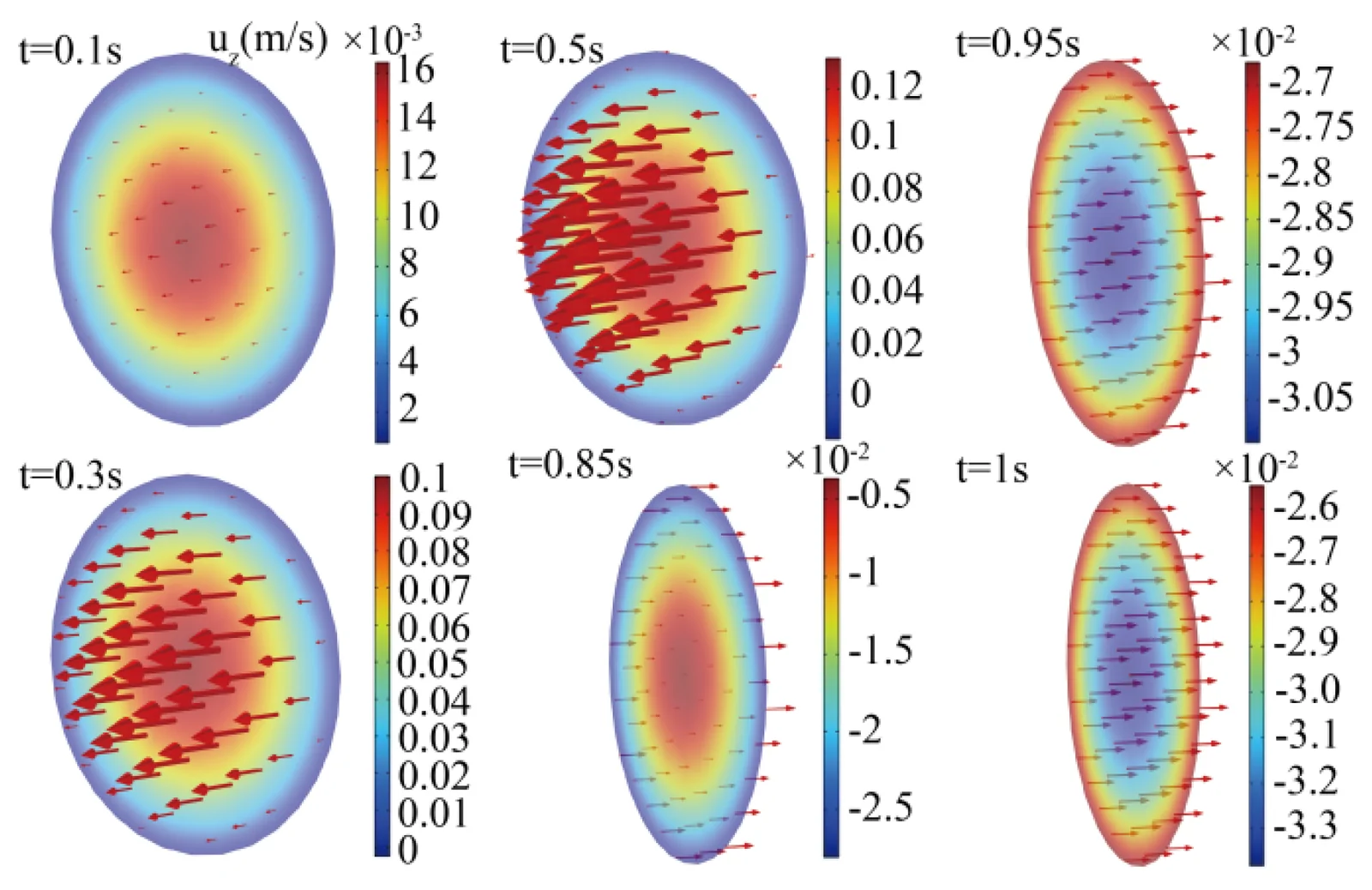
Periodic-flow vector fields for the sinusoidal wave.

Figure 17 illustrates the evolution of the flow during the first cycle under sinusoidal loading, as revealed by the flow vector field. At the initial heating stage (*t* = 0.1 s), thermal expansion of the gas in the hot reservoir establishes a positive pressure gradient, which drives a parabolic velocity profile from the hot end to the cold end. This indicates that forward Poiseuille flow is dominant. As the hot-end temperature rises, the pressure difference between the two ends increases, intensifying the forward Poiseuille flow in the central region. By *t* = 0.3 s, the temperature gradient becomes sufficiently pronounced, and a negative thermal transpiration flow emerges in the near-wall region. At *t* = 0.5 s, when the hot-end temperature reaches its maximum, the pressure difference also peaks, and the forward Poiseuille flow velocity at the centre attains its cycle maximum. During the subsequent cooling phase, the forward Poiseuille flow velocity decreases as the pressure difference diminishes, but the temperature gradient exhibits a hysteresis effect. The wall thermal transpiration flow continues to strengthen and gradually spreads toward the central region of the tube. At *t* = 0.85 s, the cold-end pressure exceeds that of the hot end, establishing a reverse pressure gradient from the cold end to the hot end. A backward Poiseuille flow begins to emerge in the central region. Simultaneously, as the cooling process weakens the temperature gradient, the thermal transpiration flow reaches its cycle peak and then gradually declines.

Near *t* = 0.95 s, the reverse pressure difference increases further, and the backward Poiseuille flow intensifies, with its velocity surpassing that of the wall thermal transpiration flow. Consequently, the entire cross-section exhibits a parabolic velocity distribution directed from the cold end to the hot end. At *t* = 1.0 s, when the temperature reaches its minimum, the backward Poiseuille flow velocity attains its cycle peak. As the next heating phase begins, this backward flow progressively decays.

### 3.3. Comparative Analysis of Thermal Non–Equilibrium Processes

Simulation results for six periodic temperature loading waveforms (rectangular, piecewise, square, Gaussian pulse, sinusoidal, and triangular) enable a cross-comparison of the peak velocities for three flow states: forward Poiseuille flow, backward Poiseuille flow, and wall thermal transpiration flow. The peak velocities of forward Poiseuille flow (Figure 6, Figure 7, Figure 8, Figure 9, Figure 10, Figure 11, Figure 12, Figure 13, Figure 14, Figure 15, Figure 16 and Figure 17) decrease in the following order: square wave, rectangular wave, piecewise wave, Gaussian pulse, sinusoidal wave, and triangular wave. For the absolute values of peak velocities in backward Poiseuille flow, the descending order is square wave, sinusoidal wave, triangular wave, rectangular wave, piecewise wave, and Gaussian pulse. Regarding the absolute values of peak thermal transpiration flow velocities, the order is square wave, sinusoidal wave, triangular wave, Gaussian pulse, rectangular wave, and piecewise wave. Based on the structural characteristics of the temperature waveforms, these six waveforms can be classified into two categories. The first category includes waveforms with distinct isothermal holding periods, such as square, rectangular, and piecewise waves. The second category consists of waveforms without isothermal holding periods, where the temperature changes continuously throughout the cycle without plateau stages. This group includes sinusoidal, triangular, and Gaussian pulse waves. Waveforms in the latter category remain in a transitional state of either heating or cooling during the entire period, and their heating and cooling rates are generally lower than those of the former category.

A comparison of peak velocities between the two waveform categories indicates that forward Poiseuille flow peak values for waveforms with isothermal holding periods (Figure 5) are consistently higher than those for waveforms without such periods. This difference is attributable to variations in heating and cooling rates. Waveforms with isothermal holding periods, particularly square and rectangular waves, demonstrate nearly instantaneous or extremely rapid heating and cooling rates during the heating phase. Such rapid heating [37] causes the gas at the hot end to expand quickly, establishing a high positive pressure gradient in a short time and thereby generating a stronger forward Poiseuille flow. In contrast, waveforms without isothermal holding periods, including sinusoidal, triangular, and Gaussian pulse waves, exhibit more gradual and continuously varying heating and cooling rates. As a result, pressure accumulation is distributed over a longer period, preventing the formation of a comparable peak pressure gradient within a short time frame. Consequently, the forward Poiseuille flow intensity is lower for these waveforms. Further analysis shows that, within the category of waveforms with isothermal holding periods, the forward Poiseuille flow peak is positively correlated with the heating and cooling rates. The square wave exhibits the highest heating and cooling rates and, correspondingly, the highest forward Poiseuille flow peak. The rectangular wave follows, while the piecewise wave, due to its slope transition during the heating phase, has an initial heating and cooling rate lower than that of the rectangular wave, resulting in a slightly reduced forward Poiseuille flow peak. Among waveforms without isothermal holding periods, the Gaussian pulse displays a lower heating rate at the early stage, which accelerates in the middle stage. Its overall heating and cooling rate is marginally higher than those of the sinusoidal and triangular waves, leading to a relatively higher forward Poiseuille flow peak. The sinusoidal and triangular waves possess similar heating and cooling rates. However, the triangular wave’s shorter residence time in the high-temperature region results in a slightly lower forward Poiseuille flow peak compared to the sinusoidal wave.

The intensity of thermal transpiration flow primarily depends on the magnitude of the temperature gradient and the duration for which it is sustained (Figure 6, Figure 7, Figure 8, Figure 9, Figure 10, Figure 11, Figure 12, Figure 13, Figure 14, Figure 15 and Figure 16). Larger temperature differences and extended residence times at elevated temperatures result in a more pronounced thermal transpiration effect, leading to higher peak velocities. Comparison of thermal transpiration flow rankings among the six waveforms demonstrates strong consistency with the equivalent residence time each waveform maintains within the higher-temperature range (approximately 350 K) throughout the cycle. Waveforms with longer residence times in this range exhibit greater thermal transpiration flow intensity. The square wave, which features the longest high-temperature isothermal plateau, produces the strongest thermal transpiration flow. Although the sinusoidal and triangular waves lack plateaus, their relatively slow curve changes result in extended residence times near the high-temperature region, yielding flow intensities between those of the square wave and the Gaussian pulse wave. The rectangular and piecewise waves possess high-temperature plateaus of limited duration, and their heating processes involve step changes or gradual transitions that inhibit full establishment of the temperature gradient. Consequently, their thermal transpiration flow intensities are lower than that of the Gaussian pulse wave.

A comparison of the peak velocity rankings for backward Poiseuille flow and thermal transpiration flow across six waveforms (Figure 6, Figure 7, Figure 8, Figure 9, Figure 10, Figure 11, Figure 12, Figure 13, Figure 14, Figure 15 and Figure 16) demonstrates a high degree of consistency. The intensity of backward Poiseuille flow is influenced by thermal transpiration flow. Thermal transpiration flow is a near-wall reverse flow driven by a temperature gradient, which transports gas from the cold end to the hot end. During the cooling phase, prior to a decrease in hot-end temperature and a relative increase in cold-end pressure, thermal transpiration flow continuously pumps gas in the reverse direction during the high-temperature plateau, resulting in additional mass accumulation at the hot end. This redistribution of mass due to thermal transpiration alters the magnitude of the reverse pressure gradient during cooling. A stronger thermal transpiration flow leads to greater mass accumulation at the hot end. As the gas at the hot end contracts, the reverse pressure difference between the two ends increases, thereby driving a more intense backward Poiseuille flow. The backward Poiseuille flow is also interconnected with the forward Poiseuille flow [38,39,40]. In terms of temporal evolution, backward Poiseuille flow represents a reversed manifestation of forward Poiseuille flow following heating, high-temperature holding, and cooling. The forward Poiseuille flow transports gas from the hot end to the cold end during heating, establishing an initial mass accumulation at the cold end. Subsequently, thermal transpiration flow further enhances cooling-induced contraction at the hot end. Therefore, the intensity of backward Poiseuille flow is determined by two factors. The baseline mass at the cold end established by forward Poiseuille flow and the additional cooling-induced contraction at the hot end resulting from thermal transpiration flow during this period.

The observed cycle-to-cycle amplification of thermal transpiration, accompanied by attenuation of forward Poiseuille flow, reveals a positive feedback loop. Increased transpiration in one cycle results in greater mass redistribution at the hot end, which enhances the reverse pressure gradient during the subsequent cooling phase. This process further strengthens transpiration in following cycles. This feedback mechanism, which has not been previously reported in the literature, indicates that the transient performance of HKCs does not necessarily approach a periodic steady state monotonically. Instead, it exhibits a gradual conditioning process over multiple cycles.

## 4. Influencing Factors for the Three Flow States

The piecewise waveform is selected for analysis to further examine the effects of heating and cooling rates, as well as high-temperature plateau residence time, on the three flow states: forward Poiseuille flow, backward Poiseuille flow, and wall thermal transpiration flow. In the first cycle, a control-variable methodology is applied, with heating time, cooling time, and high-temperature plateau residence time varied independently. Changes in the peak velocities of the three flow states are monitored to determine the influence of each parameter. The study is structured into the following four cases.

### 4.1. Simultaneous Variation in Heating and Cooling Times

**Figure 18:**
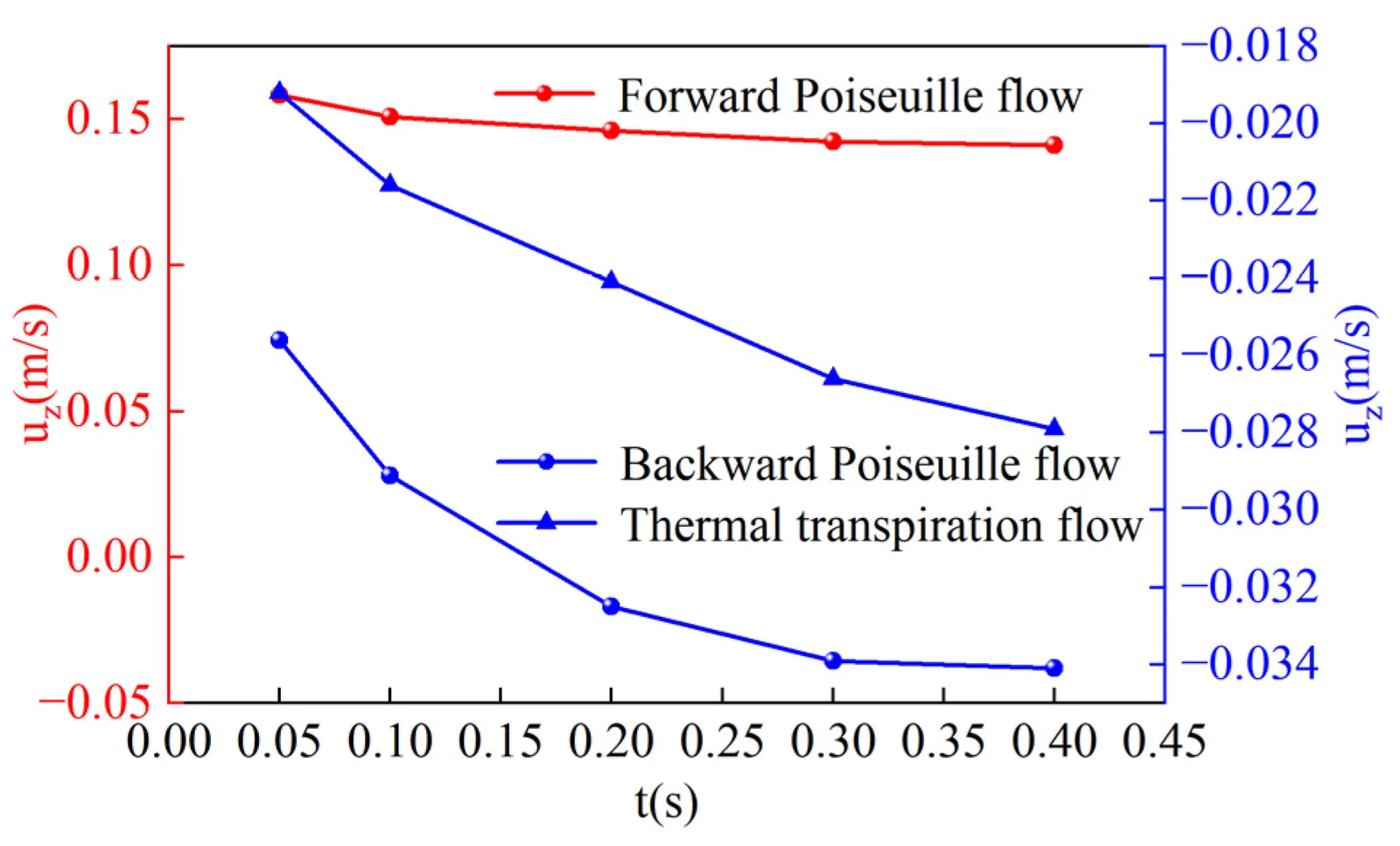
Temporal evolution of the three flow velocities with simultaneous variation in heating and cooling times.

The high-temperature plateau residence time remains constant, while the durations of the heating and cooling phases are adjusted by equal increments. The initial heating and cooling times are both 0.2 s, and four comparative parameter sets are selected. 0.05 s, 0.1 s, 0.3 s, and 0.4 s. Following one complete cycle, the peak velocities of the three flow states are presented in Table 2, and their variation trends are illustrated in Figure 18.

The peak velocity of the forward Poiseuille flow decreases significantly as heating and cooling times increase. Specifically, increasing the heating and cooling time from 0.05 s to 0.4 s results in a reduction of the peak velocity from 0.1584 m/s to 0.1410 m/s, corresponding to an approximate decrease of 11.0%. In contrast, the absolute value of the peak velocity of the backward Poiseuille flow increases with longer heating and cooling times, rising from −0.0256 m/s to −0.0341 m/s, which represents an increase of about 33.2%. Similarly, the peak velocity of the thermal transpiration flow increases from −0.0192 m/s to −0.0279 m/s, an increase of approximately 45.3%, as the heating and cooling time is extended.

These phenomena can be explained by several physical mechanisms. Increasing the heating and cooling time results in a longer residence time of the hot end near the high-temperature region. This condition favors the stable establishment of the temperature gradient and supports the sustained development of the thermal transpiration effect, which enhances the thermal transpiration flow. In contrast, a slower heating rate reduces the initial expansion intensity of the gas at the hot end, resulting in insufficient positive pressure accumulation and a subsequent weakening of the forward Poiseuille flow. The enhancement of the backward Poiseuille flow is closely associated with the disruptive influence of the thermal transpiration flow on the pressure gradient. A stronger thermal transpiration flow transports more mass in the reverse direction during the high-temperature plateau, generating a larger reverse pressure difference during the cooling phase. Furthermore, as shown in Figure 18, the amplitudes of the peak velocities for the three flow states gradually decrease with increasing heating and cooling time. This trend suggests a saturation or bottleneck effect, where further extension of the heating and cooling time produces a progressively weaker regulatory effect on the flow states once a threshold is surpassed.

### 4.2. Variation in Heating Time Only

**Figure 19:**
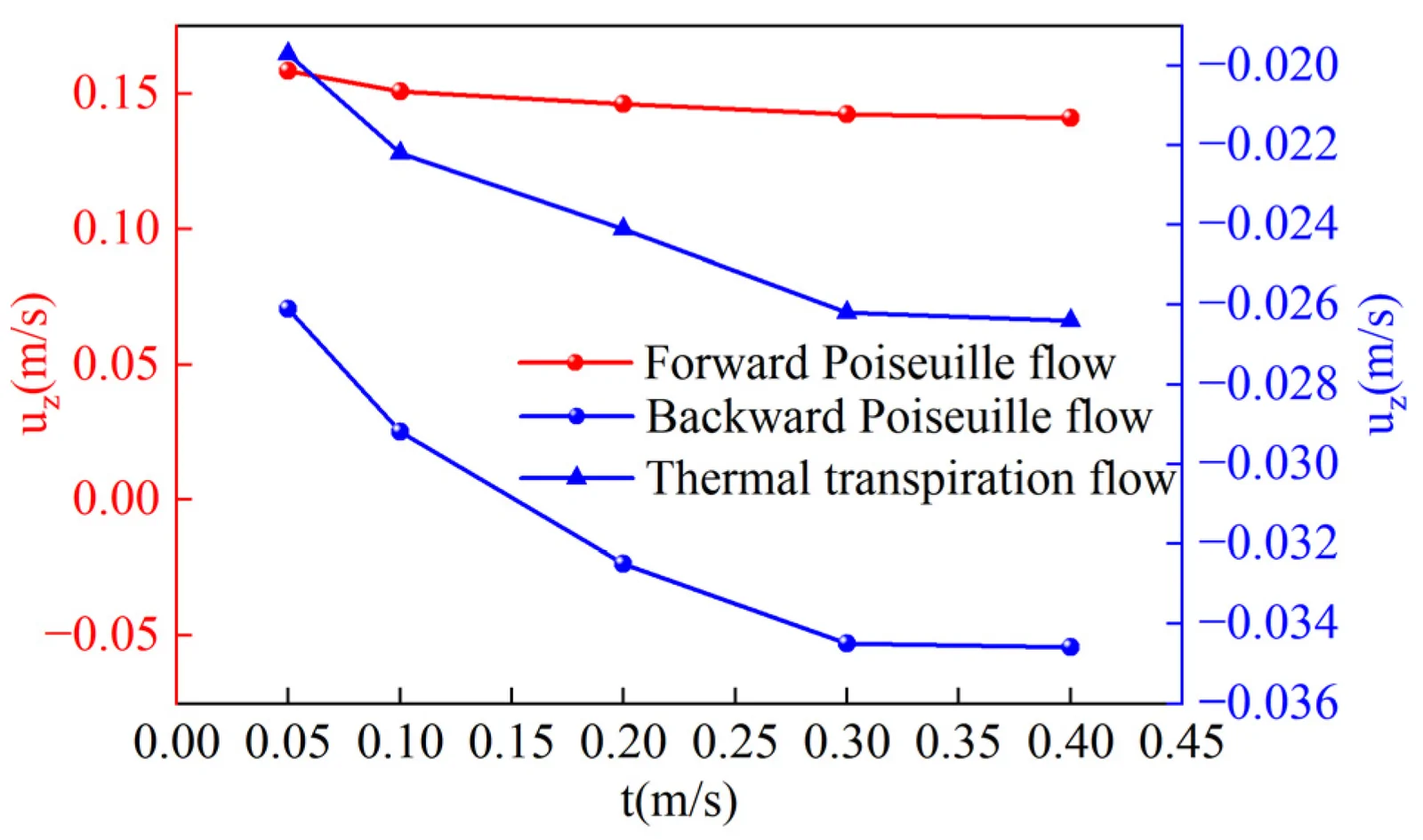
Temporal evolution of the three flow velocities with variation in heating time only.

The cooling time was maintained at the original value of 0.2 s, and the high-temperature plateau residence time was kept constant. The heating time was varied to 0.05 s, 0.1 s, 0.3 s, and 0.4 s. The corresponding results are presented in Table 3, and the trends in the three flow states are illustrated in Figure 19.

A comparison between Figure 18 and Figure 19 demonstrates that the trend in peak velocity of the forward Poiseuille flow mirrors the pattern observed when both heating and cooling times are varied simultaneously. Specifically, peak velocity decreases significantly as heating time increases. This finding indicates that the intensity of the forward Poiseuille flow is primarily governed by the heating rate. Shorter heating times and higher heating rates result in more pronounced thermal expansion of the gas at the hot end, leading to a higher peak positive pressure gradient and, consequently, a stronger forward Poiseuille flow.

The absolute value of the peak velocity of the backward Poiseuille flow increases as heating time increases, a trend that aligns with observations when both heating and cooling times are varied simultaneously. Although a longer heating time directly weakens the forward Poiseuille flow, it also extends the residence time of the hot end within the high-temperature region. This extension enhances the reverse transport capacity of the thermal transpiration flow. The increased reverse transport contributes more significantly to mass accumulation at the cold end, ultimately resulting in a stronger backward Poiseuille flow.

The peak velocity of the thermal transpiration flow increases significantly as heating time increases. This effect occurs because prolonged heating indirectly extends the residence time in the high-temperature region, thereby providing a longer developmental window for the thermal transpiration effect. Notably, similar to the case of simultaneous variations, the amplitudes of peak velocity variations for the three flow states exhibit a gradually decreasing trend with increasing heating time. This observation further confirms the presence of a bottleneck effect.

### 4.3. Variation in Cooling Time Only

**Figure 20:**
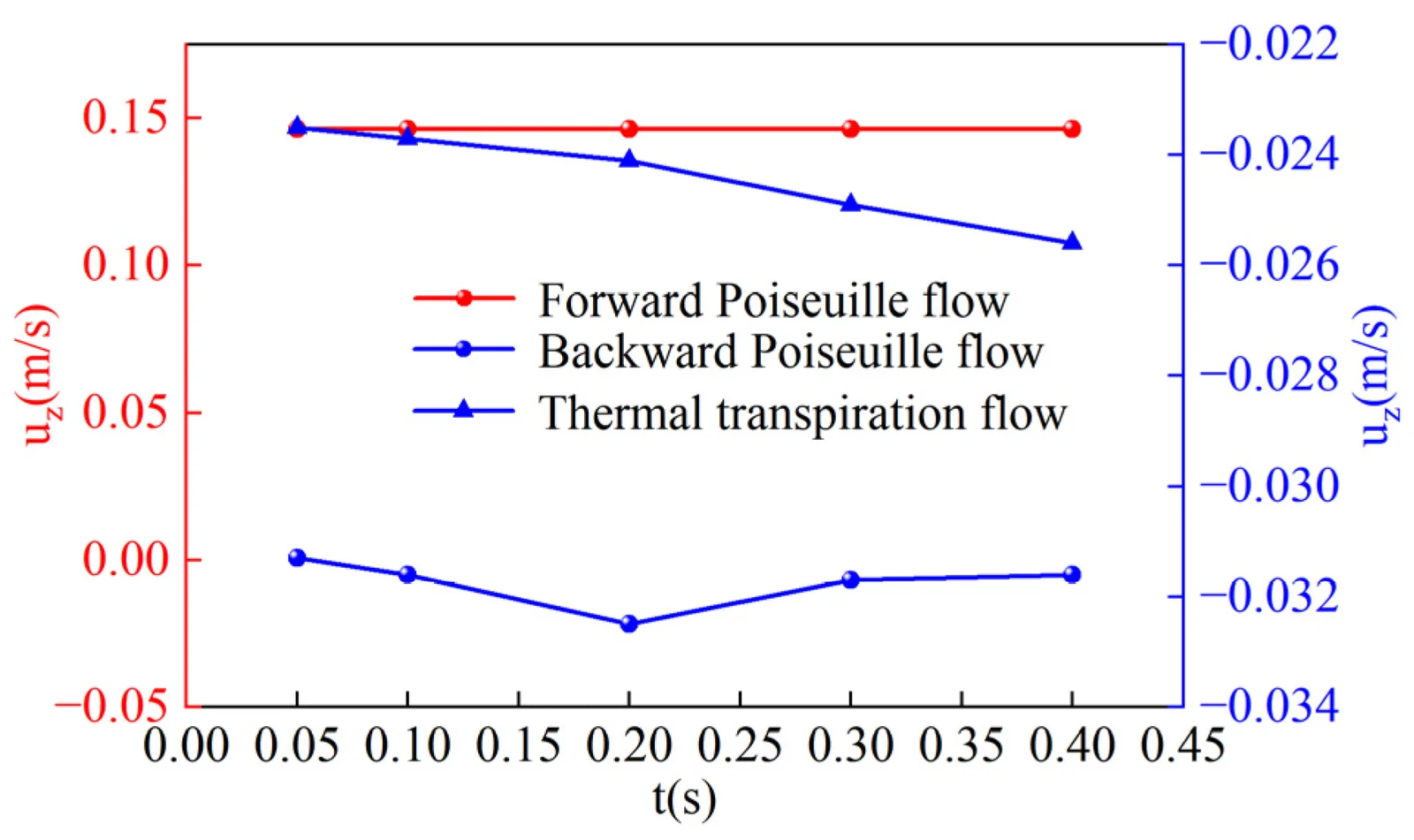
Temporal evolution of the three flow velocities with variation in cooling time only.

The heating time was maintained at 0.2 s, and the high-temperature plateau residence time was kept constant. The cooling time was varied to 0.05 s, 0.1 s, 0.3 s, and 0.4 s. The corresponding results are presented in Table 4, and the observed variation trends are illustrated in Figure 20.

Figure 20 demonstrates that the peak velocity of the forward Poiseuille flow remains stable at approximately 0.1464 m/s across all cooling times, indicating minimal sensitivity to variations in cooling duration. This finding suggests that the intensity of the forward Poiseuille flow is determined solely by the pressure accumulation established during the heating process and is independent of the cooling phase rate.

The absolute value of the peak velocity of the backward Poiseuille flow remains stable, ranging from −0.0313 m/s to −0.0325 m/s, with no monotonic trend observed. This stability suggests that the strength of the backward Poiseuille flow is not directly determined by the cooling rate. Instead, it is primarily influenced by the initial mass accumulation at the cold end, which is established by the forward Poiseuille flow. Because the heating process is identical in all cases, the mass distribution at the cold end before cooling begins remains consistent. Therefore, regardless of the cooling rate, the peak magnitude of the reverse pressure gradient exhibits minimal variation.

In contrast, the peak velocity of the thermal transpiration flow increases with longer cooling times, rising from −0.0235 m/s to −0.0256 m/s, which constitutes an increase of approximately 8.9%. This increase is attributed to the extended residence time of the hot end in the high-temperature region, which allows the thermal transpiration effect to persist longer and transport a greater amount of reverse mass. Furthermore, as illustrated in Figure 20, the amplitude of variation in the peak thermal transpiration velocity increases progressively with extended cooling time, indicating an accelerating growth trend. This finding is consistent with the high sensitivity of thermal transpiration flow to residence time at elevated temperatures.

### 4.4. Variation in High-Temperature Plateau Residence Time Only

**Figure 21:**
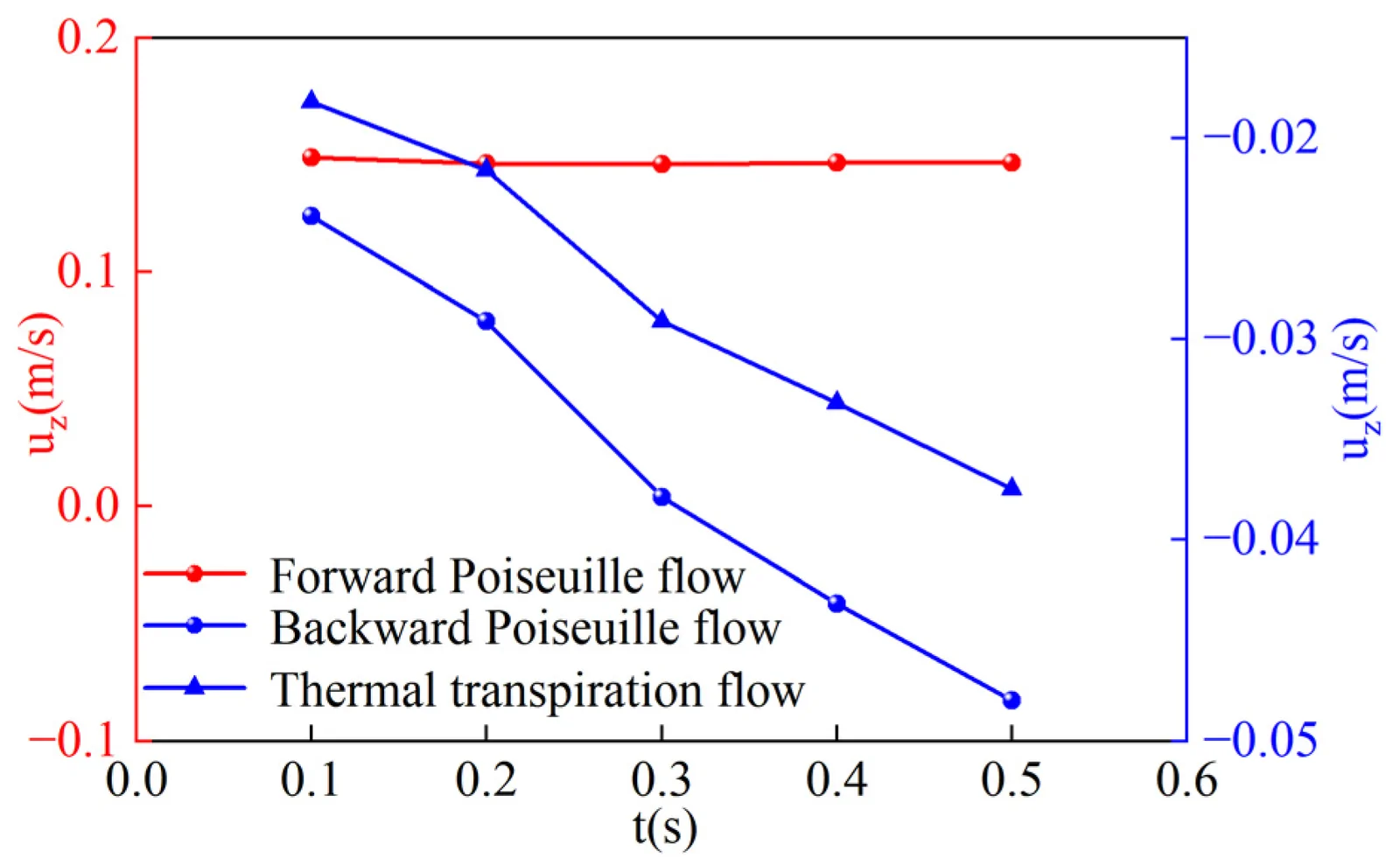
Temporal evolution of the three flow velocities with variation in high-temperature plateau residence time.

The heating and cooling times are fixed at 0.2 s, while the residence time on the high-temperature isothermal plateau is varied across five values. 0.1 s, 0.2 s, 0.3 s, 0.4 s, and 0.5 s. The results are presented in Table 5, and the corresponding trends are illustrated in Figure 21.

Figure 21 demonstrates that the peak velocity of the forward Poiseuille flow remains largely unchanged as the high-temperature plateau residence time increases, stabilizing at approximately 0.146 m/s. This observation suggests that the plateau length exerts minimal influence on the initial establishment of the positive pressure gradient, since the peak forward Poiseuille flow occurs at the end of the heating phase (approximately 0.4–0.5 s), before the plateau duration becomes relevant.

The absolute value of the peak velocity of the backward Poiseuille flow increases substantially as the high-temperature plateau residence time increases, rising from −0.0239 m/s at 0.1 s to −0.0480 m/s at 0.5 s, which constitutes an increase of more than 100%. This increase occurs because, during a longer plateau, thermal transpiration flow continuously transports gas from the cold end to the hot end, thereby compensating for some of the mass lost at the hot end. As a result, during the cooling phase, the contraction effect of the gas at the hot end intensifies, generating a larger reverse pressure gradient and facilitating the full development of the backward Poiseuille flow. Furthermore, the growth rate of the backward Poiseuille flow accelerates with extended plateau duration, demonstrating a pronounced nonlinear characteristic.

The peak velocity of the thermal transpiration flow increases substantially with longer high-temperature plateau residence times, rising from −0.0182 m/s to −0.0375 m/s, which represents an increase of approximately 106%. This finding directly confirms the significant enhancing effect of the high-temperature isothermal region on the thermal transpiration phenomenon. A longer plateau duration sustains the temperature gradient for an extended period, thereby strengthening the cumulative effect of thermal transpiration. As shown in Figure 21, both the backward Poiseuille flow and the thermal transpiration flow demonstrate consistent monotonic increases in peak velocity as plateau residence time extends, and their growth trends are closely aligned. These results further demonstrate the driving influence of thermal transpiration flow in promoting backward Poiseuille flow.

The four sets of control-variable experiments systematically demonstrate the distinct effects of heating and cooling durations, as well as high-temperature plateau residence time, on the three flow states. The primary findings are as follows. The forward Poiseuille flow is primarily determined by the heating rate and remains unaffected by the cooling rate and plateau duration. The backward Poiseuille flow is indirectly influenced by the plateau duration and the intensity of thermal transpiration flow, while it exhibits insensitivity to the cooling rate. The thermal transpiration flow shows a positive correlation with the effective residence time in the high-temperature [41,42,43] region and is further enhanced by extended heating and cooling durations.

The duration of the high-temperature plateau does not affect the peak forward Poiseuille flow, as this peak occurs before the plateau. In contrast, the plateau duration has a nonlinear amplifying effect on both thermal transpiration and backward Poiseuille flow. The observation that both peaks more than double when the plateau is extended from 0.1 to 0.5 s suggests that cumulative mass transport during the isothermal hold induces a memory effect [44,45] on the subsequent cooling phase. This effect arises from the finite time required for thermal transpiration to redistribute mass along the entire channel length. Longer plateaus allow this redistribution to approach a quasi-equilibrium state, which maximizes the reverse pressure difference during cooling.

## 5. Conclusions

This study conducts a systematic numerical simulation to investigate the transient thermo-fluid-mass coupling behavior of a hydrogen Knudsen compressor subjected to periodic temperature loading. The transient evolution characteristics and competitive mechanisms among forward Poiseuille flow, backward Poiseuille flow, and wall thermal transpiration flow are analyzed for six representative temperature waveforms. rectangular, piecewise, square, Gaussian pulse, triangular, and sinusoidal. The principal findings are summarized below.

(1) Throughout the cyclic variation of the six temperature waveforms, the hydrogen Knudsen compressor completes a full operational cycle. During the heating phase, forward Poiseuille flow is predominant. As the system enters the high-temperature period, thermal transpiration flow intensifies, which weakens the forward pressure gradient. In the cooling phase, the pressure gradient reverses, resulting in the dominance of backward Poiseuille flow. As the number of cycles increases, thermal transpiration flow becomes stronger and persistently disrupts the pressure gradient. This process reduces the forward pressure difference that can be re-established in subsequent heating phases and increases the reverse pressure difference during cooling phases. Consequently, the forward Poiseuille flow velocity decreases with each cycle, while the backward Poiseuille flow velocity increases. Over the entire sequence, the maximum forward Poiseuille flow velocity is observed near the heating peak of the first cycle, whereas the peak backward Poiseuille flow and wall thermal transpiration flow are both observed near the cooling trough of the final cycle.

(2) During the high-temperature plateau, thermal transpiration flow continuously transports gas from the cold end to the hot end, resulting in additional mass accumulation at the hot end. As the hot-end temperature subsequently decreases, the contraction effect intensifies, which influences the magnitude of the reverse pressure gradient during the cooling phase. The isothermal plateau suppresses Poiseuille flow. Within this plateau segment, thermal transpiration flow persists in the reverse direction and gradually disrupts the established pressure gradient. Consequently, the effective development duration of Poiseuille flow is significantly shorter than its nominal existence time. A high-temperature plateau promotes the establishment and enhancement of thermal transpiration flow, while a low-temperature plateau reduces the temperature gradient and inhibits thermal transpiration development. However, due to the rapid heating rates associated with waveforms containing plateaus, the forward Poiseuille flow peak values are consistently higher than those observed in continuous waveforms without plateaus. The intensity of thermal transpiration flow is positively correlated with the equivalent residence time in the high-temperature region. The square wave, characterized by the highest heating and cooling rates and the longest high-temperature residence time, produces the largest peak velocities for all three flow components.

(3) The heating rate primarily determines the intensity of the forward Poiseuille flow, while the cooling rate and high-temperature plateau duration have minimal impact. Prolonged heating time can reduce the peak forward Poiseuille flow velocity by up to 11.0%. Increasing the heating time, cooling time, or high-temperature plateau residence time enhances the thermal transpiration flow, with the plateau duration alone contributing to an increase of up to 106% in its peak velocity. The backward Poiseuille flow is influenced by both the plateau duration and the thermal transpiration flow, with its intensity increasing by over 100%, although it remains largely unaffected by the cooling rate. Saturation effects are evident across different flow states, as the amplitude of peak velocity variations decreases with longer heating and cooling times. The thermal transpiration flow, in particular, demonstrates an accelerating growth trend with extended cooling time, indicating high sensitivity to the high-temperature residence period.

The local Knudsen number near the cold inlet marginally exceeds 0.1 under transient conditions, indicating that the slip-boundary Navier–Stokes model used in this study possesses inherent physical limitations in local quantitative accuracy during flow transitions. Future research will employ the Direct Simulation Monte Carlo method to validate high-Knudsen-number transient local zones and to quantify error margins associated with the continuum assumption in the present scenario. All slip-boundary computations utilize a tangential momentum accommodation coefficient of *α_t_* = 0.9, which aligns with the widely accepted value for hydrogen–silicon interfaces as reported in Ref. [21]. Thermal transpiration in the slip regime is highly sensitive to the wall molecular reflection model. The absolute ranking of thermal transpiration peaks among the smooth continuous waveforms (Gaussian, sinusoidal, and triangular) should be regarded as a relative trend under the specific choice of *α_t_* = 0.9, rather than a universally conclusive order. Nonetheless, qualitative comparisons between plateau-type and smooth-continuous-type waveform groups, including heating-rate dominance for forward Poiseuille flow and plateau-time amplification for thermal transpiration, remain robust across the entire *α_t_* range considered. Experimental calibration or DSMC simulations are recommended in future studies to determine the actual *α_t_* for specific surface conditions and to further refine quantitative predictions.

## Figures and Tables

**Table 1:** Key structural parameters of the hydrogen Knudsen compressor.

Parameter	Size
The length *l* of the microchannel	30 μm
The length *L* of the container	30 μm
The diameter *d* of microchannel	0.2 μm
The diameter *D* of the container	60 μm
Silicon thickness *t_si_*	1.2 μm
Cold container temperature *T*_1_	300 K
Hot container temperature *T*_2_	300–350 K
gaseous medium	H_2_

**Table 2:** Effect of heating and cooling times on flow states.

Heating and Cooling Times (s)	Peak Velocity of Forward Poiseuille Flow (m/s)	Peak Velocity of Backward Poiseuille Flow (m/s)	Peak Thermal Transpiration Flow Velocity (m/s)	Heating and Cooling Times (s)
0.05	0.1584	−0.0256	−0.0192	0.05
0.1	0.1509	−0.0291	−0.0216	0.1
0.2	0.1464	−0.0325	−0.0241	0.2
0.3	0.1423	−0.0339	−0.0266	0.3
0.4	0.1410	−0.0341	−0.0279	0.4

**Table 3:** Effect of heating time on flow states.

Heating Times (s)	Peak Velocity of Forward Poiseuille Flow (m/s)	Peak Velocity of Backward Poiseuille Flow (m/s)	Peak Thermal Transpiration Flow Velocity (m/s)	Heating Times (s)
0.05	0.1584	−0.0261	−0.0197	0.05
0.1	0.1509	−0.0292	−0.0222	0.1
0.2	0.1464	−0.0325	−0.0241	0.2
0.3	0.1423	−0.0345	−0.0262	0.3
0.4	0.1410	−0.0346	−0.0264	0.4

**Table 4:** Effect of cooling time on flow states.

Cooling Times (s)	Peak Velocity of Forward Poiseuille Flow (m/s)	Peak Velocity of Backward Poiseuille Flow (m/s)	Peak Thermal Transpiration Flow Velocity (m/s)	Cooling Times (s)
0.05	0.1464	−0.0313	−0.0235	0.05
0.1	0.1464	−0.0316	−0.0237	0.1
0.2	0.1464	−0.0325	−0.0241	0.2
0.3	0.1464	−0.0317	−0.0249	0.3
0.4	0.1464	−0.0316	−0.0256	0.4

**Table 5:** Effect of high-temperature plateau residence time on flow states.

High-Temperature Plateau Residence Time (s)	Peak Velocity of Forward Poiseuille Flow (m/s)	Peak Velocity of Backward Poiseuille Flow (m/s)	Peak Thermal Transpiration Flow Velocity (m/s)	High-Temperature Plateau Residence Time (s)
0.1	0.149	−0.0239	−0.0182	0.1
0.2	0.1464	−0.0325	−0.0241	0.2
0.3	0.1459	−0.0379	−0.0291	0.3
0.4	0.1467	−0.0432	−0.0332	0.4
0.5	0.1468	−0.048	−0.0375	0.5

## Data Availability

The data that support the findings of this study are available from the corresponding author upon reasonable request.

## Abbreviations

The following abbreviations are used in this manuscript:

HKC	hydrogen Knudsen compressor
NS	Navier–Stokes
